# Intercorrelations of Chlorinated Paraffins, Dechloranes, and Legacy Persistent Organic Pollutants in 10 Species of Marine Mammals from Norway, in Light of Dietary Niche

**DOI:** 10.1021/acs.est.4c02625

**Published:** 2024-08-09

**Authors:** Clare Andvik, Eve Jourdain, Anders Borgen, Jan Ludvig Lyche, Richard Karoliussen, Tore Haug, Katrine Borgå

**Affiliations:** † Department of Biosciences, University of Oslo, Pb 1066 Blindern, Oslo NO-0316, Norway; ‡ Norwegian Orca Survey, Breivikveien 10, Andenes NO-8480, Norway; § Department of Environmental Chemistry, NILU: The Climate and Environmental Research Institute, Pb 100, Kjeller NO-2027, Norway; ∥ Department of Food Safety and Infection Biology, Norwegian University of Life Sciences, Pb 5003, Ås NO-1432, Norway; ⊥ Institute of Marine Research, Fram Centre, Pb 6606 Stakkevollan, Tromsø NO-9296, Norway

**Keywords:** stable isotopes, emerging contaminants, northern bottlenose whale, white-beaked dolphin, humpback whale, fin whale, harbor seal, harbor porpoise, isotopic niche, top predator, biomagnification, bioaccumulation

## Abstract

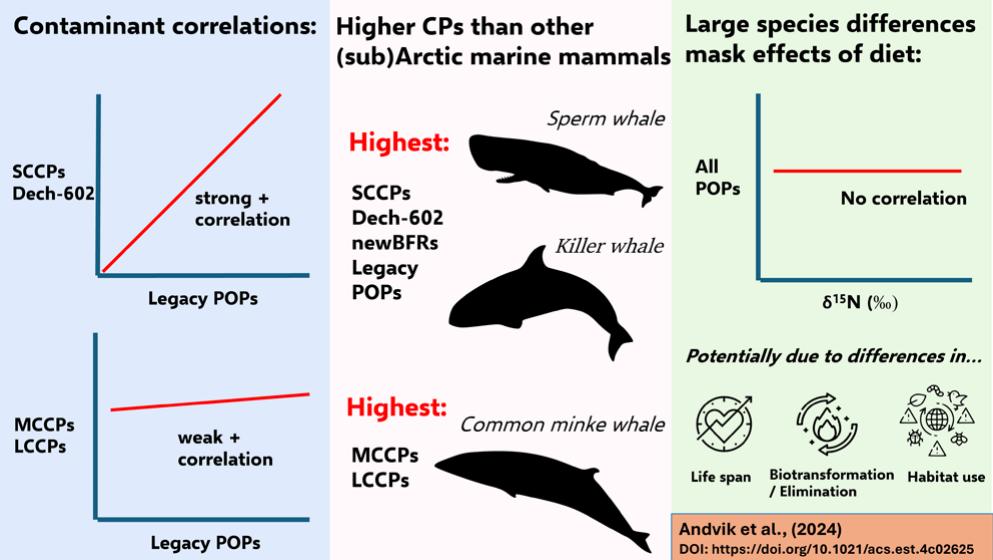

Short-, medium-, and long-chain chlorinated paraffins (CPs) (SCCPs, MCCPs, and LCCPs) and dechloranes are chemicals of emerging concern; however, little is known of their bioaccumulative potential compared to legacy contaminants in marine mammals. Here, we analyzed SCCPs, MCCPs, LCCPs, 7 dechloranes, 4 emerging brominated flame retardants, and 64 legacy contaminants, including polychlorinated biphenyls (PCBs), in the blubber of 46 individual marine mammals, representing 10 species, from Norway. Dietary niche was modeled based on stable isotopes of nitrogen and carbon in the skin/muscle to assess the contaminant accumulation in relation to diet. SCCPs and dechlorane-602 were strongly positively correlated with legacy contaminants and highest in killer (*Orcinus orca*) and sperm (*Physeter macrocephalus*) whales (median SCCPs: 160 ng/g lw; 230 ng/g lw and median dechlorane-602: 3.8 ng/g lw; 2.0 ng/g lw, respectively). In contrast, MCCPs and LCCPs were only weakly correlated to recalcitrant legacy contaminants and were highest in common minke whales (*Balaenoptera acutorostrata*; median MCCPs: 480 ng/g lw and LCCPs: 240 ng/g lw). The total contaminant load in all species was dominated by PCBs and legacy chlorinated pesticides (63–98%), and MCCPs dominated the total CP load (42–68%, except 11% in the long-finned pilot whale *Globicephala melas*). Surprisingly, we found no relation between contaminant concentrations and dietary niche, suggesting that other large species differences may be masking effects of diet such as lifespan or biotransformation and elimination capacities. CP and dechlorane concentrations were higher than in other marine mammals from the (sub)Arctic, and they were present in a killer whale neonate, indicating bioaccumulative properties and a potential for maternal transfer in these predominantly unregulated chemicals.

## Abstract 2

Short-chain-chlorinated paraffins (CPs) and dechloranes positively correlated with recalcitrant legacy contaminants to a stronger extent than medium- or long-chain CPs across marine mammals, and CP concentrations were surprisingly unrelated to dietary niche.

## 1 Introduction

Persistent organic pollutants (POPs) occurrence in Arctic wildlife indicate contaminants’ mobility and bioaccumulation potential in an environment with few local sources.^1,2^ Occurrence in top predators, such as marine mammals, can additionally be used as a proxy for marine ecosystem health, as marine mammals often present the highest concentrations of contaminants in an environment due to high trophic positions, thick blubber layers, and long lifespans.^3,4^ Of growing concern are contaminants of emerging (Arctic) concern (CECs); replacement chemicals for regulated “legacy” POPs that are not subject to international regulations but still have the potential to harm human and animal health, or regulated chemicals with increased presence in the Arctic.^2,5^ Examples of CECs include chlorinated paraffins (CPs) and dechloranes.

CPs and dechloranes are chlorinated compounds with extensive global use and production as flame retardants and/or plasticizers.^6,7^ CPs are grouped based on the alkane carbon chain lengths in their products: short-chain CPs (SCCPs, C_10–13_), medium-chain CPs (MCCPs, C_14–17_), and long-chain CPs (LCCPs, C_18–30_). Homologue groups are further defined by the number of chlorine substituents for each carbon chain length. The most common types of dechloranes include two stereoisomers of Dechlorane Plus (DP) (C_18_H_12_Cl_12_), dechlorane-602 (C_14_H_4_Cl_12_O), dechlorane-603 (C_17_H_8_Cl_12_), and dechlorane-604 (C_13_H_4_Br_4_Cl_6_). SCCPs have been globally regulated under the Stockholm Convention since 2017,^8^ and in 2021, MCCPs were placed on the European Candidate List of substances of very high concern^9^ and have been proposed for regulation under the Stockholm Convention.^10^ DP was listed under the Stockholm Convention in 2023,^11^ but LCCPs and other dechloranes are unregulated internationally. CPs and dechloranes are highly lipophilic, with indications of biomagnification in aquatic food webs.^7,12−14^ SCCPs and MCCPs have been shown to positively correlate with trophic level in an Arctic food web spanning from cod to polar bears (*Ursus maritimus*),^15,16^ as well as a food web study in the Great Lakes,^12^ and from mollusks to marine mammals in the South China Sea,^17^ indicating bioaccumulative and biomagnification properties. Data on LCCPs are scarcer but have been detected in marine mammals.^18^ Correlations to trophic level are weaker for MCCPs than SCCPs, and it has been suggested that larger CP molecules resist mass transfer in the uptake process.^15,19^ However, few studies directly compare how the different CP classes and dechloranes occur in predators compared with legacy POPs. As it is difficult to compare CP studies and laboratories due to challenges in analytical procedures and a lack of commercially available chemical standards and reference materials,^20,21^ it is of greater importance to compare their behavior with other chemicals analyzed on the same individuals.

A range of studies have detected both legacy and emerging contaminants in Arctic marine mammals in Svalbard, Greenland, the Baltic, Canada, and Scandinavia, e.g. CPs,^18,19,22−24^ dechloranes,^25,26^ and legacy POPs.^1,2,27−29^ Monitoring studies from the Norwegian coast, however, have focused on sediments, mussels, and fish,^30,31^ with occurrence in top predators restricted to occasional studies on killer whale (*Orcinus orca*)^32−35^ and harbor porpoise (*Phocoena phocoena*).^35,36^ To date, there have been no studies of CPs or dechloranes in any marine mammals from coastal Norway.

Dietary niche and trophic position is a strong determinant for biomagnification of recalcitrant substances,^37^ and contaminant concentrations can vary in marine mammals based on diet.^32,34,38^ The diet of marine mammals can be estimated using stable isotopes of nitrogen (δ^15^N), which increase in a predictable manner from food source to consumer and indicate relative trophic position,^39,40^ and carbon (δ^13^C), which indicate feeding habitat due to differing carbon sources at the base of the food chain, with the lowest δ^13^C values in species feeding on pelagic prey offshore and highest values in species feeding on benthic prey in coastal waters.^41−43^ Integrating these two metrics delineates a species’ isotopic niche, which can be used to map and statistically compare organisms’ feeding ecology and habitat use.^44,45^ Despite the wide range of marine mammal species occurring in Norway, only limited studies of isotopic niche have been conducted, e.g., in the Norwegian High Arctic as opposed to coastal Norway.^46^

In this study, we aimed to investigate the occurrence of CPs and dechloranes, compared to legacy contaminants, in 10 marine mammal species of differing diets. We analyzed samples from nine species stranded on the coast of Norway and one species harvested in the Barents Sea to (1) quantify the isotopic niche; (2) quantify concentrations of CPs, dechloranes, legacy POPs, and a selection of unregulated brominated flame retardants (BFRs); and (3) investigate contaminant intercorrelations and patterns. Factors potentially explaining concentrations and patterns, such as diet, species, sex, age class, and carcass decomposition state, were also explored.

## 2 Materials and Methods

### 2.1 Sampling

Blubber, skin, and, where possible, muscle samples were collected from 32 individual marine mammals of nine species that were stranded in 2015–2020 along the Norwegian coast, including specimens from a mass stranding of 17 whales of seven species that occurred in Northern Norway in March 2020.^47^ In addition, samples were collected from 14 common minke whales (*Balaenoptera acutorostrata*; hereafter minke whale) from the 2019 annual commercial harvest in the Barents Sea (Figure S1).

The stranded specimens comprise eight killer whales, including one neonate, seven sperm whales (*Physeter macrocephalus*), six harbor porpoises, four humpback whales (*Megaptera novaeangliae*), three harbor seals (*Phoca vitulina*), two fin whales (*Balaenoptera physalus*), one long-finned pilot whale (*Globicephala melas*), one northern bottlenose whale (*Hyperoodon ampullatus*), and one white-beaked dolphin (*Lagenorhynchus albirostris*). Sample collection of stranded marine mammals was coordinated by the Norwegian Orca Survey (NOS) and conducted by NOS scientists or local volunteers. Sample collection of harvested minke whales was conducted by the Norwegian Institute of Marine Research and described in detail elsewhere.^28^ Blubber samples were wrapped in aluminum foil and stored at −20 °C upon sampling, during transport, and at the University of Oslo until analysis. Where possible, sex and age class (neonate, subadult, and adult) of each individual were determined by morphological characteristics or identification of sex organs as described, for example, for killer whales^48^ and minke whales.^49^ The decomposition state of each stranded individual was coded based on established protocols where 2 = freshly deceased and no bloating, 3 = moderately decomposed with mild to moderate bloating, and 4 = advanced decomposition with major bloating/organs beyond recognition (Table 1).^50^ We had different sample sizes for the various analyses due to limitations in the sample size or tissue type (Tables 1; S1; S2).

**Table 1:** Median (Range) Concentrations of Short-, Medium-, and Long-Chain CPs (SCCPs, MCCPs, and LCCPs, Respectively) and Dechloranes in Marine Mammals of Different Species, Age Class, and Sex Sampled from Norway 2015–2020 (ng/g lw)^a^

species	*n*	age; sex	year	code	lipid %	SCCPs	MCCPs	LCCPs	Dech-602	DP *syn*	DP *anti*
common minke whale (*Balaenoptera acutorostrata*)	5	adult; female	2019	2	77 (64–86.3)	42 (<LOD–110)	500 (<LOD–1400)	120 (60–290)	0.27 (0.09–0.72)	<LOD	<LOD
	5	adult; male	2019	2	58 (45–76)	140 (110–260)	1300 (450–1900)	200 (40–290)	0.52 (0.12–1.3)	0.13 (<LOD–0.16)	0.22 (<LOD–0.28)
	4	subadult; female	2019	2	63 (23–89)	46 (<LOD–58)	240 (<LOD–450)	480 (370–1100)	0.34 (0.04–1.5)	<LOD	<LOD
	**14**	all	2019	2	64 (23–89)	69 (<LOD–260)	480 (<LOD–1900)	240 (40–1100)	0.36 (0.04–1.5)	0.07 (<LOD–0.16)	0.13 (<LOD–0.28)
killer whale (*Orcinus orca*)	3	adult; female	2016–2017	3	64 (52–85)	170 (160–210)	440 (360–660)	89 (41–110)	7.2 (5.2–14)	0.12 (0.06–0.17)	0.15 (<LOD–0.21)
	2	adult; male	2016–2017	2–3	(35–64)	(130–200)	(420–2000)	(74–620)	(1.1–79)	(<LOD–0.35)	(<LOD–0.94)
	1	adult; UNK	2015	4	66	300	480	170	1.6	<LOD	<LOD
	1	neonate; male	2017	2	65	110	290	69	0.36	0.079	0.17
	1	subadult; male	2016	2	41	110	320	120	2.5	0.045	0.074
	**8**	all	2015–2017	2–4	64 (35–85)	160 (110–300)	430 (290–2000)	99 (41–620)	3.8 (0.36–79)	0.097 (<LOD–0.35)	0.16 (<LOD–0.94)
sperm whale (*Physeter macrocephalus*)	1	adult; female	2020	3	33	230	330	160	1.4	<LOD	<LOD
	5	adult; male	2018–2020	2–4	22 (11–83)	340 (87–1600)	510 (220–2400)	72 (31–700)	2.1 (1.4–2.5)	1.0 (<LOD–4.2)	1.3 (<LOD–5.2)
	1	subadult; male	2020	3	54	160	280	37	1.3	<LOD	<LOD
	**7**	all	2018–2020	2–4	33 (11–83)	230 (87–1600)	330 (220–2400)	72 (31–700)	2.0 (1.3–2.5)	0.13 (<LOD–4.2)	0.22 (<LOD–5.2)
harbor porpoise (*Phocoena phocoena*)	2	adult; female	2020	2	(4–97)	(90–720)	(56–100)	(47–98)	(0.80–1.7)	<LOD	<LOD
	4	UNK	2020	2–4	80 (77–86)	53 (37–67)	69 (<LOD–110)	25 (<LOD–37)	0.70 (0.12–1.1)	<LOD	0.081 <LOD–0.099
	**6**	all	2020	2–4	80 (4–97)	51 (37–720)	70 (<LOD–110)	35 (<LOD–98)	0.89 (0.12–1.7)	<LOD	0.093 <LOD–0.099
humpback whale (*Megaptera novaeangliae*)	1	adult; female	2020	3	74	60	110	37	0.078	<LOD	<LOD
	1	adult; male	2020	4	77	26	3.5	11	0.029	<LOD	0.16
	1	subadult; female	2020	3	74	91	310	65	0.063	<LOD	<LOD
	1	UNK	2019	3	67	66	180	18	0.044	<LOD	<LOD
	**4**	all	2019–2020	3–4	74 (67–77)	63 (26–91)	150 (3.5–310)	28 (11–65)	0.054 (0.029–0.078)	<LOD	0.099 (<LOD–0.16)
fin whale (*Balaenoptera physalus*)	**1**	adult; female	2020	3	75	<LOD	<LOD	<LOD	0.061	<LOD	<LOD
	**1**	adult; UNK	2020	2	74	95	130	31	1.6	<LOD	<LOD
harbor seal (*Phoca vitulina*)	**1**	adult; female	2020	2	62	66	5.0	2.0	1.2	0.083	0.23
	**1**	UNK	2017	2	87	39	89	11	1.7	<LOD	<LOD
long-finned pilot whale (*Globicephala melas*)	**1**	subadult; UNK	2020	4	59	20	<LOD	12	0.14	<LOD	<LOD
northern bottlenose whale (*Hyperoodon ampullatus*)	**1**	UNK; male	2020	4	77	39	140	37	0.48	<LOD	<LOD
white-beaked Dolphin (*Lagenorhynchus albirostris*)	**1**	UNK	2020	3	76	84	250	11	0.27	<LOD	<LOD

a UNK = unknown. NA = not analyzed. LOD = limit of detection (values given in Table S1) code = decomposition code.^50^

### 2.2 Stable Isotope Analysis

δ^15^N and δ^13^C were analyzed at the CLIPT Stable Isotope Laboratory at the University of Oslo. Analysis was conducted on the freeze-dried and homogenized skin (*n* = 37) and/or muscle (*n* = 30). The skin and muscle were both targeted because some individuals only had one of the two tissues available for sampling, and the muscle indicates diet over a longer time period than the skin.^51^ δ^13^C values were determined from a lipid-extracted aliquot with a 2:1 chloroform/methanol solution to correct for the low δ^13^C found in the lipid fraction of an organism.^43,52^ δ^15^N values were determined from the non–lipid extracted samples due to the unpredictable changes in δ^15^N values following lipid extraction.^53,54^ The full method and quality assurance are described previously for killer whales,^55^ and internal references and calibrations in the present study were within acceptable ranges, with standard deviations for δ^15^N 0.02‰ and δ^13^C 0.04‰.

δ^15^N and δ^13^C results in the 10 adult minke whale skin and muscle are presented in MacKenzie et al.^46^ and for seven killer whale skin and four killer whale muscle in Andvik et al.^33^ δ^15^N and δ^13^C results for 20 skin samples and 16 muscle samples from sperm whale, long-finned pilot whale, harbor seal, harbor porpoise, white-beaked dolphin, northern bottlenose whale, humpback whale, and fin whale are presented for the first time in the present paper.

### 2.3 CP and Dechlorane Analysis

SCCPs, MCCPs, LCCPs, and seven dechloranes were quantified in the blubber of 46 individuals at The Climate and Environmental Research Institute NILU Kjeller. The full method and quality assurance are described in detail in the Supporting Information. Briefly, the method utilizes sodium sulfate in homogenization, lipid removal with sulfuric acid and cleaning with silica and sodium sulfate. Fractionation by Florisil cleanup was conducted to correct for the large amount of polychlorinated biphenyls (PCBs) in the samples. The presence of toxaphenes, chlordanes, and nonachlor, which cannot be removed by Florisil cleanup, can sometimes interfere to a minor extent in the traces of some of the CP congener groups. However, for all samples in the present study no interfering peaks were present and no further corrections during quantification were necessary. Gas and liquid chromatography with mass spectrometry was utilized to analyze for the compounds, and quantification was based on the deconvolution method developed by Bogdal et al.^56^ The limit of detection (LOD), limit of quantification, mean chlorination degree (%), and the % found in all samples can be found in Table S3.

### 2.4 Legacy Organochlorine and BFR Analysis

We analyzed 50 legacy organochlorines (OCs; 34 PCB congeners and 16 organochlorinated pesticides) and 14 legacy BFRs, including 13 polybrominated diphenyl ethers (PBDEs), and four emerging BFRs in blubber of 42 individuals at the Laboratory of Environmental Toxicology at the Norwegian University of Life Sciences (MT-laboratory NMBU), Ås, Norway. We used a multicomponent method first described in 1978,^57^ which utilizes approximately 0.5 g of tissue, extraction by cyclohexane and acetone, and lipid removal by sulfuric acid. The method, lab accreditations, and quality assurance are described in detail for a range of compounds and biological matrices elsewhere^58,59^ and are the same as previously described for killer whales.^32^ Certified reference materials (CRM350, CRM598, and CRM2525), internal reference material (contaminated seal blubber and MTref01) and blanks were within approved ranges for the current analyses, and a complete list of analyzed compounds is available in Table S4, along with limits of detection, internal standard recoveries, and % found in all samples.

Legacy contaminant concentrations in blubber for the 10 adult minke whales and eight killer whales are reported elsewhere,^28,33^ and results for the remaining 24 individuals (sperm whale, long-finned pilot whale, harbor seal, harbor porpoise, white-beaked dolphin, northern bottlenose whale, humpback whale, and fin whale) are presented for the first time in the present paper.

### 2.5 Data Treatment

Data were treated using R (v. 4.2.3).^60^ Isotopic niche widths were calculated separately for the skin and muscle using a Bayesian stable isotope standard ellipse area, corrected for the small sample size (SEA_C_) using the package SIBER.^61^ Niche widths were statistically compared, and the proportion of overlap relative to non-overlapping areas between each cluster calculated using Bayesian standard ellipse areas (SEA_B_), generated using 10^6^ posterior draws for each cluster. Ellipses, isotopic niche widths, and proportional overlaps were only calculated for species which had at least *n* = 3 for both the skin and muscle: the minke whale, killer whale, harbor porpoise, and sperm whale, and ellipses represent 40% prediction areas, which are equivalent to 40% of the data regardless of sample size.^61^ Isotopic baselines, and associated isotopic values in consumers, can vary by year and geographic region.^62,63^ We assume any isotopic baseline variation to be low compared to the dietary variation in the present study, as all individuals were sampled in a limited geographic area and monitoring studies from the Norwegian coast have shown very similar yearly isotopic signatures in blue mussels (*Mytilus edulis*), a reliable baseline indicator species.^31^

For contaminants, we used a function in R to replace values below the LOD with a random number between 0 and the LOD, assuming a beta distribution (α = 5 and β = 1) to retain the pattern of the data set.^64^ This consisted of 452 values and 15% of the data set for legacy OCs and BFRs, 13 values and 9.4% of the data set for CPs, 0 samples for dechlorane-602, and 65 values and 70% of the samples for the DP isomers combined. Substitutions were not conducted on the other dechloranes as all but one individual were <LOD. When reporting contaminant concentrations in tables and graphs, we included all analyzed contaminants, including imputed values. When conducting statistical and multivariate analyses, however, we included only those contaminants found in over 70% of the samples to ensure that the data set did not have a high proportion of substituted numbers. This excluded PCB-56, heptachlor, BDE-196-202, -206, -207, -208, -209, the four emerging BFRs pentabromotoluene (PBT), pentabromoethylbenzene (PBEB), 3-dibromopropyl-2,4,6-tribromophenyl ether (DPTE), and hexabromobenzene (HBB) and all dechloranes except dechlorane-602.

Principle component analysis (PCA) was used to visualize the interrelations among the log-10 transformed concentrations of all contaminants (wet weight), the patterns of all contaminants, and the patterns of CP homologue groups between the species. In the concentrations PCA, lipid % was included as a covariable in the visualization, and variables were unscaled to visualize both the covariances of variables and differences in contaminant concentrations. For the pattern PCAs, variables were scaled to zero mean and unit standard deviation and normalized to total contaminant concentrations (for the PCA including all contaminants) or total CP concentrations (for the CP homologue group PCA). For the concentrations and pattern PCAs, 42 individuals were plotted, excluding the four subadult minke whales for which legacy OC and BFR data were not available. PCBs were divided into metabolic groups according to the presence of vicinal H atoms and Cl-substitution in the ortho-meta and meta–para positions (groupings described in Figure 2). PCB metabolic groups I, II, and V are not readily metabolized in marine mammals and are dominated by more highly chlorinated hexa- and hepta-CBs, while groups III and IV are metabolized by the cytochrome P450 (CYP) 1A1 (CYP1A1) enzyme and dominated by the less chlorinated tri- and tetra-chlorinated congeners.^65,66^ For the PCA of patterns of CP homologue groups, individuals were only included that had concentrations >LOD for SCCPs, MCCPs, and LCCPs and thus had homologue data available. This excluded the single pilot whale, two of the 14 minke whales (one adult female and one subadult female), one of the two fin whales, one of the four humpback whales, and two of the six harbor porpoises. We also excluded the four subadult female minke whales from the CP homologue group PCA due to a very high proportion of LCCPs but are included in the Supporting Information (Figure S2). Only homologue groups found in at least one individual were included in the PCA. This excluded the SCCPs C_10_Cl_4_, C_11_Cl_4_, C_11_Cl_11–13_, the MCCPs C_12_Cl_4_, C_12_Cl_11_, C_13_Cl_4_, C_13_Cl_9–12_, C_14_Cl_10_, C_14_Cl_4_, C_15_Cl_9–10_, C_16_Cl_4–5_, C_16_Cl_8–10_, C_17_Cl_4–10_, and all the LCCPs of carbon chain 22 and above. This represents 90 homologue groups and 59% of the total number of homologue groups analyzed.

Redundancy analysis (RDA) was used to determine significant associations between the contaminant response variables, and the explanatory variables species, lipid %, age class, sex, decomposition code, δ^15^N in the skin, δ^15^N in the muscle, δ^13^C in the skin, and δ^13^C in the muscle. Because the effect of species was assumed to be large, models were run with and without species as an explanatory variable to explore any masked associations. Due to some missing explanatory variables for some individuals, six RDAs were conducted on concentrations and six on patterns with and without species as an explanatory variable and with differing sample sizes to explore the associations (Table S5). Due to strong correlations between δ^15^N values in the skin and muscle and δ^13^C values in the skin and muscle, only skin or muscle values were used in a model not both. Significant explanatory variables were determined as variables present in the best model, determined by a forward model selection from the null to full model, followed by a Monte Carlo permutation test (1000 unrestricted permutations).

We used Spearman’s rank correlation to test the relationship between δ^15^N and SCCPs, MCCPs, LCCPs, dechlorane-602, and PCB-153, as well as between contaminants, using the Benjamini & Hochberg false discovery rate method to adjust for *p*-value inflation by multiple testing.

## 3 Results and Discussion

### 3.1 Dietary Niche

**Figure 1:**
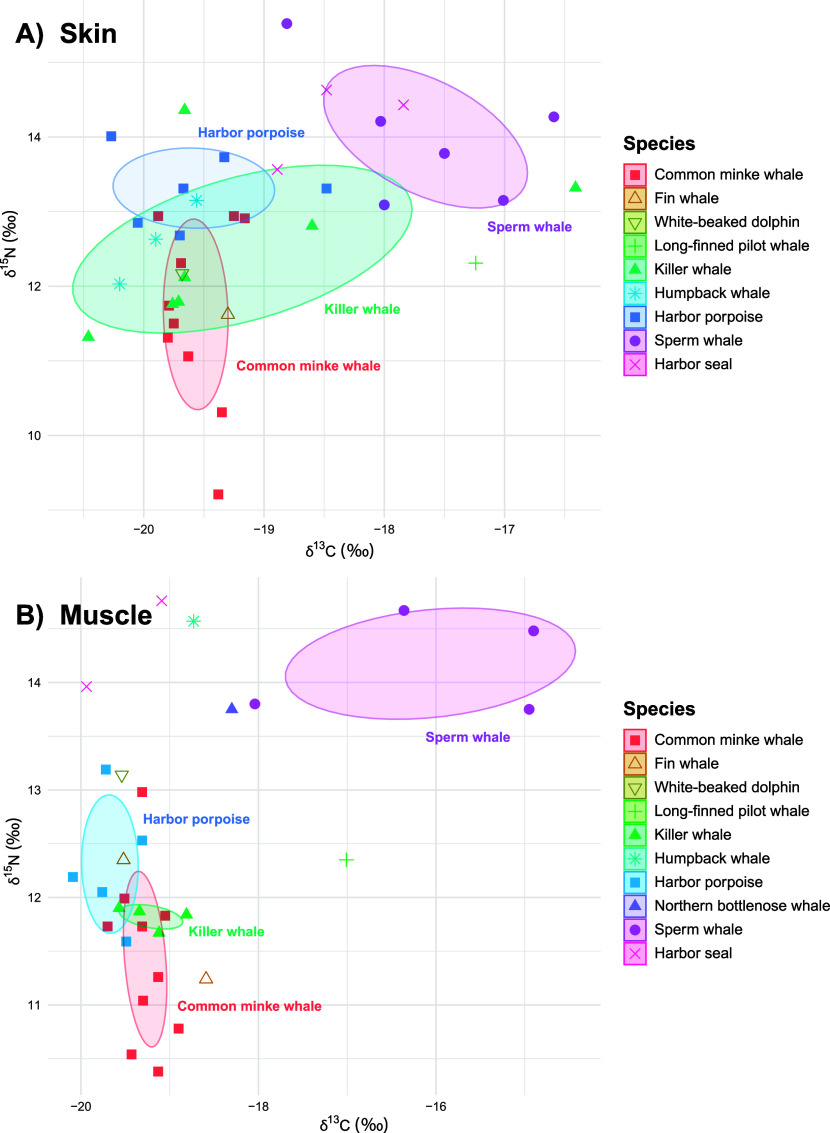
δ^13^C and δ^15^N signature in the (A) skin (*n* = 38) and (B) muscle (*n* = 31) of marine mammals from Norway 2015–2020. Species are represented as unique colors and shapes, and ellipses are 40% prediction areas for each species.

In general, our data corroborate what is known about the feeding habits of these species. The minke whale had the lowest median δ^15^N and δ^13^C values of all species (Figure 1; Table S2), which suggest feeding on low to midtrophic pelagic prey. The minke whale also had a wide range of δ^15^N values (a range of 3.7‰ in the skin and 2.6‰ in the muscle, which is roughly equivalent to one trophic level change^40^) indicating a breadth of prey types, which align with other observational and dietary studies of minke whales feeding opportunistically on a wide range of pelagic fish and crustaceans across multiple trophic levels/regions of different isotopic baselines.^67−69^ Fin and humpback whales had similar median δ^15^N and δ^13^C values to minke whales (Figure 1; Table S2), indicating similarities in feeding habits, and both have been observed feeding on pelagic schooling fish and crustaceans,^70^ although with humpbacks also extending to higher trophic level prey,^71^ which is supported by the singularly high δ^15^N value in muscles for a humpback whale in the present study (Figure 1; Table S2). Humpback and killer whales have been observed feeding on the same prey patch of herring (*Clupea harengus*) in the winter,^72^ and all of the humpback skin samples fall within the killer whale dietary niche. The killer whales had one of the lowest median δ^15^N values, although they had the largest isotopic niche width in the skin, indicating a breadth of prey types (Figure 1; Table S6). Killer whales in Norway are known to feed on not only a range of fish prey, predominantly herring, but also marine mammal prey.^73^ One of the killer whales (ID OO4) was found with seal hair in their throat and had the highest δ^15^N value of all the adult killer whales. White-beaked dolphins and harbor porpoises in Norway had similar δ^15^N values, which were intermediate compared to the other species, and low δ^13^C values, confirming an assumption that they are mid-trophic level species, opportunistically feeding on small pelagic fish.^74,75^ The sperm whale had the highest median δ^15^N and δ^13^C values of all species (Figure 1; Table S2), which aligns with a recorded diet of higher trophic level benthic prey, such as large cephalopods.^70,76,77^ The isotopic niche of the sperm whales, nonoverlapping with the other, primarily pelagic, isotopic niches of the killer whales, harbor porpoise, and minke whale, indicate a difference in feeding preferences and habitat for this species. Interestingly, one of the sperm whales in the present study (ID SW7) was female and had stable isotope values similar to the males, despite female sperm whales not being known to migrate to northern latitudes to feed.^78^ The long-finned pilot whale and northern bottlenose whales are also known to feed on deep-sea benthic species^79,80^ and had similarly high δ^15^N and/or high δ^13^C values to sperm whales (Figure 1; Table S2). Two of the three harbor seal skin samples also fell within the dietary niche ellipses for sperm whales. Harbor seals in Norway are known to feed primarily on the semi-pelagic saithe (*Pollachius virens*) and other benthic and pelagic fish,^81^ and the high δ^15^N values in the present study could suggest a dominance of higher trophic prey.

For all species except the sperm whales, the niche width in the muscle was significantly narrower than in the skin, and for all of the species, the proportional overlap between the two tissues was high (Table S6). The tissue-dependent isotopic turnover rate in many species is unknown, but isotopic values in the muscle are assumed to have a slower turnover rate than in the skin and be representative of a longer period.^51^ Our isotopic results suggest that the minke whale, killer whale, and harbor porpoise have short-term variations in feeding preference, as indicated by wide niches in the skin, but longer-term stability, as indicated by narrower niches in the muscle. As individuals from each species were sampled in different years, seasons, and locations, the variability in the short term can be due to each individual opportunistically taking advantage of different food sources that average out predictably on the long term. It should be noted the sample size for the muscle is lower than the skin, and uncertainties may also be present due to degradation of tissues in the stranded animals, which are known to affect isotopic values of different tissues and species in unpredictable ways.^82−84^ Nevertheless, the majority of individuals (28 of 46) were assigned decomposition code 2, indicating no degradation. This isotope data can thus give an indication of dietary niches for species where fresh samples are otherwise unavailable as well as allow the study of the relationship between pollutant concentrations and diet.

### 3.2 General Occurrence of all Contaminants

PCBs and chlorinated pesticides dominated the total contaminant load for all species, making up a total proportion of between 63% (minke whale) and 98% (long-finned pilot whale) (Figure S3). PCBs-118, -138, -153, -170, -187, and *p*,*p*′-DDE dominated these subgroups. The highest contribution of CPs were found in minke whale (23%), humpback whale (18%), fin whale (14%), and northern bottlenose whale (13%), albeit proportions were minimal compared to the legacy POPs (Figure S3). Similarly, the prevalence of emerging BFRs was small (0.001–0.01% of total contaminant load) compared to legacy BFRs (0.9–2.8% of total contaminant load) (Figure S3). The dominance of legacy contaminants over emerging contaminants is also found elsewhere, such as from the Baltics,^23^ and is likely reflective of the longer time frame in which legacy POPs have been produced and emitted into the environment compared to emerging POPs, as well as potentially higher persistence, biomagnification potential, and maternal transfer rates of legacy POPs. Despite regulations, there has been only slight general decreasing trends evident for legacy POPs in Arctic marine mammal species since 1980,^27,85−87^ and in some instances no change or increasing trends due to local effects of climate change remobilizing legacy POPs from the cryosphere.^88^

MCCPs dominated the CP profiles in all species (42–68%) except the long-finned pilot whale (11%), which instead had higher proportions of SCCPs (56%), followed by LCCPs (33%) (Table 1; Figure S4). A dominance of MCCPs may be a reflection of the increased use of MCCPs following restrictions of SCCPs, with the time period of the present study (2015–2020) closely mirroring the time frame of reduced SCCPs use in the early 2010s, and a final listing under the Stockholm Convention in 2017.^8^ Dechlorane-602 was the only dechlorane type detected in all species and in higher concentrations than the DP isomers, which was found almost exclusively in killer and sperm whales (Table 1). Lower concentrations of DPs vs dechlorane-602 could similarly reflect decreased use of DPs, with production volumes plummeting since 2011,^89^ in advance of its restriction under the Stockholm Convention in 2023.^11^

The emerging BFRs, PBT and HBB, were detected in 48% and 55% of samples, respectively, with the highest concentrations in the male killer whales and harbor porpoise (Table S1). PBEB and DPTE were not found in any sample (Table S3).

### 3.3 Comparing among Contaminants

#### 3.3.1 Contaminant Intercorrelations

**Figure 2:**
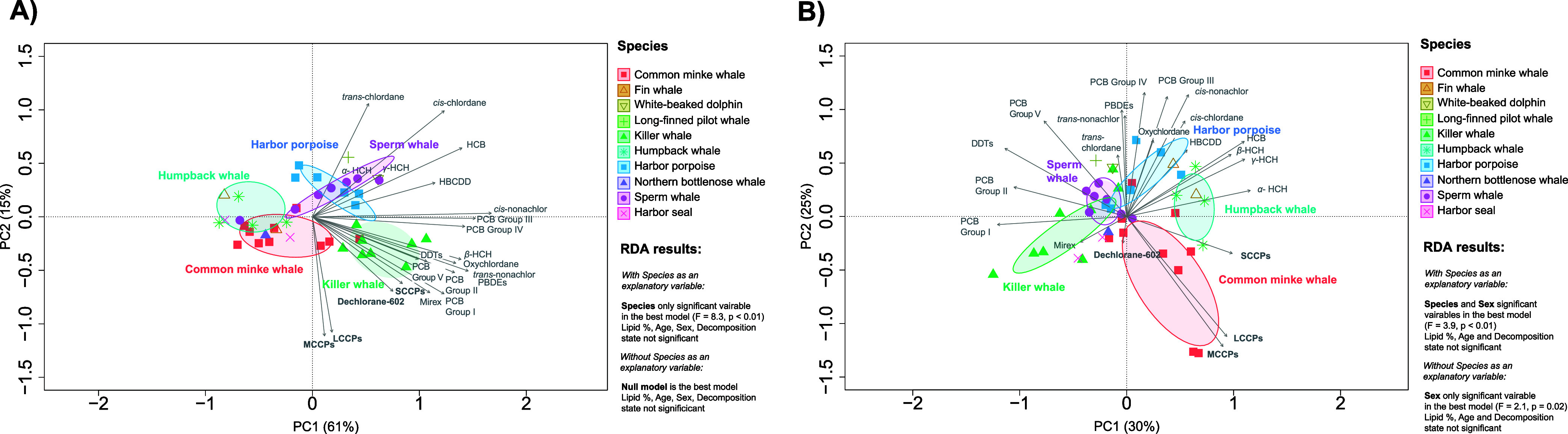
PCA biplot based on occurrence of legacy and emerging contaminants in blubber of 10 species of marine mammal from Norway 2015–2020 (*n* = 42), and results from the corresponding RDA (A) concentrations (ng/g ww) with lipid included as a co-variable. (B) Patterns, with variables normalized to total contaminant concentrations and scaled to zero mean and unit standard deviation. Response loadings are represented as gray arrows, and the species as unique colors and symbols. The ellipses represent 40% prediction areas for each species for the multivariate normal distribution. The percentages of the total variation explained by PC1 and PC2 are given in brackets on each axis. PCB group I is the sum of PCB-153, -180, -183, -187, -189, -194, -196, -199, -206, and -209 (no vicinal H-atoms); PCB group II is the sum of PCB-47-99, -114, -128, -137, -138, and -170 (vicinal H-atoms only in ortho–meta positions and ≥2 Cl in ortho-positions); PCB group III is the sum of PCB-28, -66, -74, -105, -118, -156, and -157 (vicinal H-atoms only in ortho–meta positions and <2 Cl in ortho-positions); PCB group IV is the sum of PCB-31, -52, -56, -87, -101, -110, -136, and -141 (vicinal H-atoms in meta–para positions and ≤2 Cl in ortho-positions); PCB group V is the sum of PCB-149 and -151 (vicinal H-atoms in meta–para positions and >2 Cl in *ortho*-positions). DDT is the sum of p,p′-DDE, o,p′-DDD, p,p′-DDD, o,p′-DDT, and p,p′-DDT. PBDEs are the sum of BDE-28, -47, -99, -100, -153, -154, -183, -196, -202, -207, -208, and -209.

SCCPs and dechlorane-602 had high positive correlations to legacy contaminants known to be highly persistent and resistant to metabolism in marine mammals, such as PCB metabolic groups I, II, and V and the sum of DDTs (Figure 2A; Table S7). Mirex, a pesticide and flame retardant, was also positively correlated with both SCCPs and dechlorane-602, as well as the PBDEs, suggesting that dechlorane-602 (which was produced as a replacement for mirex and deca-BDEs) behaves similarly to its replacements in marine mammals (Figure 2A, Table S7). In contrast, MCCPs and LCCPs were only very weakly positively correlated to the more persistent contaminant groups (Figure 2A; Table S7). These results suggest a different bioaccumulative potential of these emerging contaminants in marine mammals, which can be influenced by the toxicodynamics of the chemicals in organisms. While both lower trophic magnification factors (TMFs; biomagnification across several trophic levels) and biomagnification factors (BMFs; biomagnification across one trophic level from prey to predator) have been observed in MCCPs as opposed to SCCPs in a Lake Ontario food web,^12^ the opposite was observed in a South China food web, including marine mammals,^17^ indicating that biomagnification may vary between habitats/organisms. Despite the present study indicating lower bioaccumulative potential of MCCPs and LCCPs than SCCPs, it should be acknowledged that they can break down to shorter chained CPs and other potentially toxic compounds throughout their lifetime,^90^ and thus can still constitute a risk to organisms.

#### 3.3.2 Large Differences between Species Not Due to Diet

We observed large differences in the concentrations and patterns of different contaminant types in the species analyzed, and when the species type was included as an explanatory variable in the RDA models, it dominated in explanatory power (Figure 2; detailed model summaries are given in Table S5). Species differences did not, however, appear to be due to diet, with δ^15^N and δ^13^C values nonsignificant in almost all models, both with and without the species type as an explanatory variable and with and without an interaction term between δ^15^N and δ^13^C (Table S5).

**Figure 3:**
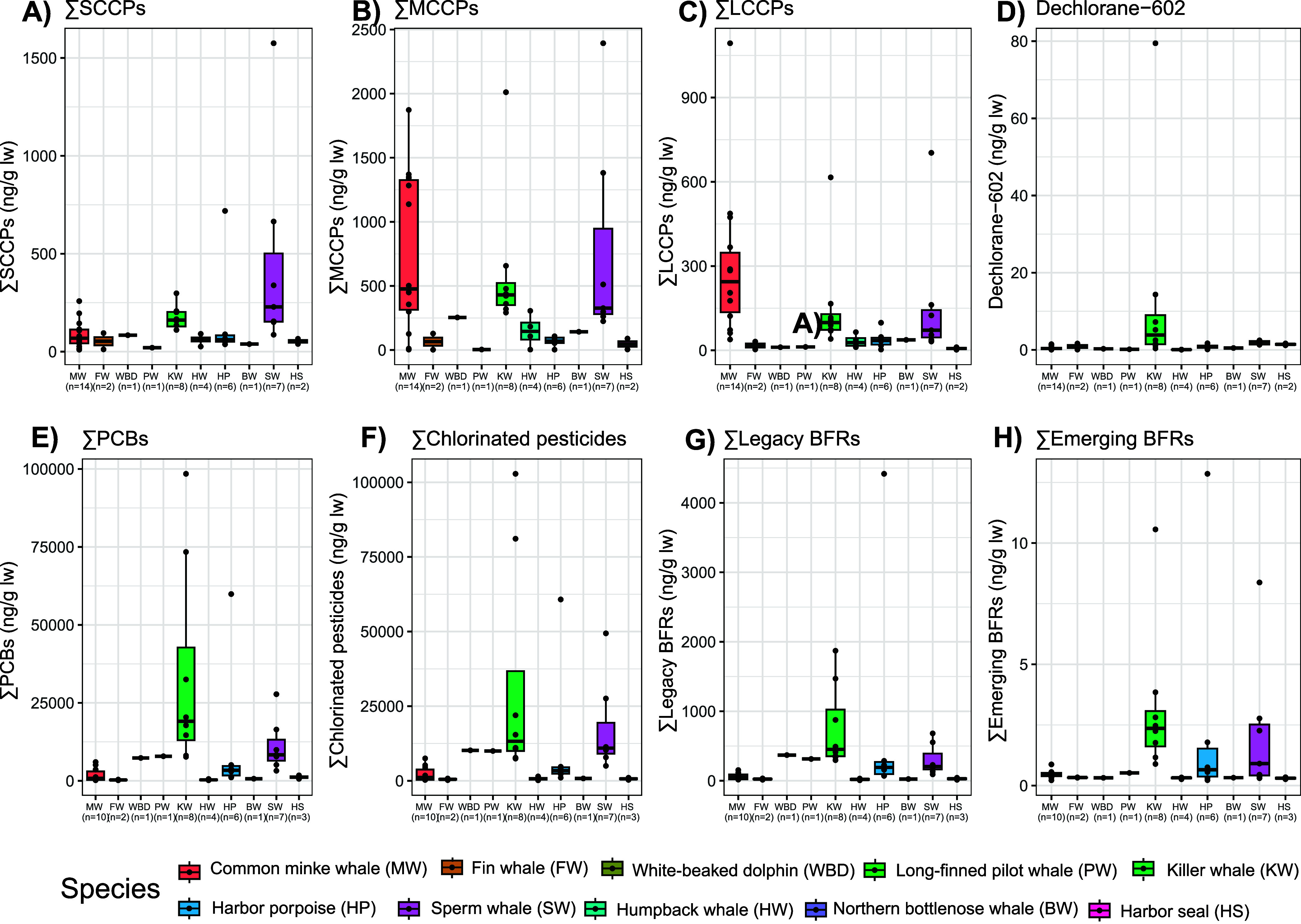
Concentrations (ng/g lw) of (A) ΣSCCPs (B) ΣMCCPs, (C) ΣLCCPs, (D) dechlorane-602, (E) ΣPCBs, (F) ΣChlorinated pesticides, (G) ΣLegacy BFRs, and (H) ΣEmerging BFRs in blubber of 10 species of marine mammal from Norway 2015–2020 (*n* = 46). Species are ordered on the *x* axis by increasing mean δ^15^N values in the skin and follow the same order as in the legend. Note different scales on *y* axis. ΣPCBs = sum of PCB-28, -31, -47, -52, -56, -66, -74, -87, -99, -101, -105, -110, -114, -118, -128, -136, -137, -138, -141, -149, -151, -153, -156, -157, -170, -180, -183, -187, -189, -194, -196, -199, -206, and -209; ΣChlorinated pesticides = sum of hexachlorobenzene (HCB), p,p′-DDE, o,p′-DDD, p,p′-DDD, o,p′-DDT, p,p′-DDT, heotachlor, oxychlordane, trans-chlordane, cis-chlordane, trans-nonachlor, cis-nonachlor, α-HCH, β-HCH, γ-HCH, and mirex; ΣLegacy BFRs = sum of hexabromocyclododecane (HBCDD), BDE-28, -47, -99, -100, -153, -154, -183, -196, -202, -207, -208 and -209; ΣEmerging BFRs = sum of pentabromotoluene (PBT), hexabromobenzene (HBB), pentabromoethylbenzene (PBEB), and 3-dibromopropyl-2,4,6-tribromophenyl ether (DPTE).

**Figure 4:**
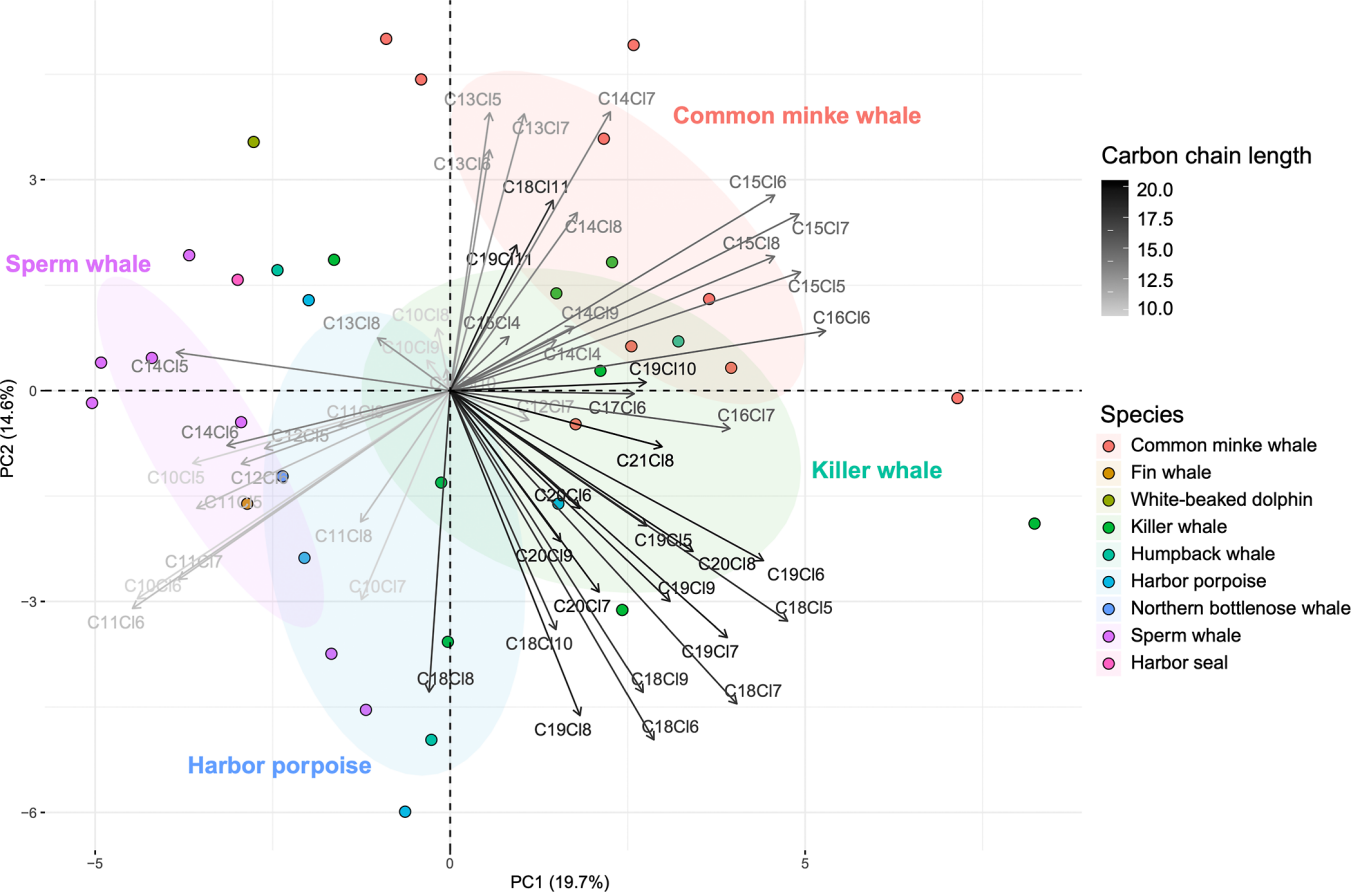
PCA biplot of the patterns of homologue groups of short-, medium-, and long-chain CPs in blubber in nine species of marine mammal from Norway 2015–2020 (*n* = 35). Response loadings are represented by arrows, colored from light gray to black by increasing carbon chain length. Unique colors represent each species are represented by unique colors. The ellipses represent 40% prediction areas for each species for multivariate normal distribution. The percentage of the total variation explained by PC1 and PC2 are given in brackets on each axis.

We found a small effect of δ^13^C values in the skin on the pattern of contaminants, but the explanatory power was at least six times lower than when the model included species (Table S5). Nevertheless, we found that higher δ^13^C values in the benthic-feeding sperm whales were associated with higher proportions of SCCPs (Table S5), which is corroborated in our other analyses. Sperm whales had the highest concentrations of SCCPs across all species (Table 1, Figure 3), and these individuals, as well as the benthic-feeding northern bottlenose whale, also had higher proportions of shorter chained and less chlorinated CP homologues (Figures 4; S5). The pilot whale, also a benthic-feeding species, had the highest proportion of SCCPs to the sum of CPs of the analyzed species (Figure S3). In contrast, the pelagic feeding killer whales, with lower δ^13^C values, had a higher proportion of CPs with longer carbon chains (Figure 4) and higher chlorination (Figure S5). A higher proportion of SCCPs, and less chlorinated CPs, can suggest a higher proportion of SCCPs in the benthic environment, leading to higher exposure in these species. Similar patterns have been observed elsewhere, such as SCCPs with fewer chlorine atoms in sediment samples as opposed to aquatic biota in the Norwegian Arctic,^16^ a higher percentage of SCCPs in benthic organisms than pelagic fish in a lake from China,^91^ and a review article concluding that benthic organisms have a higher accumulation potential to SCCPs than planktonic species.^92^

The general lack of explanatory effect of trophic level on contaminant concentrations and patterns was corroborated by no correlation being found between δ^15^N values and SCCPs, MCCPs, LCCPs, dechlorane-602, or PCB-153 concentrations (Figure S6). δ^15^N values, furthermore, did not explain contaminant concentrations or patterns in a subset of the data of just legacy POPs, despite the link between diet and contaminant concentrations being well established in marine mammals.^32,34,93^

However, there appears to be positive correlations between δ^15^N values and contaminant concentrations within some species (Figure S6). For killer whales, if the neonate is excluded (due to assumed enriched δ^15^N values from its mother’s milk), we found strong positive correlations between δ^15^N in the skin and SCCPs, MCCPs, and PCB-153 (rho = 0.5, 0.7, and 0.8, respectively), and weaker positive correlations to LCCPs and dechlorane-602 (rho = 0.1 and 0.4, respectively) (Figure S6). Similarly, we found a small effect of higher δ^15^N values in the muscle associated with higher relative contributions of SCCPs and legacy POPs when minke whales dominated the sample size (Table S5) but no effect on concentrations. Sample sizes within other species were too small for further comparisons.

Due to correlations being found between contaminants and δ^15^N within species but not between species, the large contaminant variation is likely due to other differences between the species that mask the effects of diet.

#### 3.3.3 Other Species Characteristics Explaining Contaminant Differences

The large effect of species in our models, to the almost complete exclusion of other explanatory variables, indicates that there are other, unaccounted for, species characteristics explaining the observed intraspecific differences in contamination concentrations and patterns (Figure 2; Table S5).

Life history traits, such as lifespan, are one likely explanation. We found the highest concentrations of SCCPs, dechlorane-602, and legacy contaminants in sperm whales and killer whales (Tables 1; S1; Figure 3), as well as the largest proportions of more persistent bioaccumulative pollutants, such as PCB metabolic groups I, II, and V, and ΣDDTs (Figure 2). Male killer whales can live beyond 40 years and females 80 years,^94^ and sperm whales have similarly long lifespans of approximately 65 years.^95^ In contrast, harbor porpoise and harbor seals, each with higher average δ^15^N values than killer whales but low concentrations of contaminants, have average lifespans of 8–10 years and 25–30 years, respectively.^74,96^ While we were unable to age the individuals beyond the broad age classes of adult/subadult/neonate, given the large differences in species lifespan it is likely that the adult killer and sperm whales were older than the adult harbor porpoise and seals (e.g., killer whales are categorized as adults rather than subadults at approximately 11 years of age; more than the upper range of a harbor porpoise lifespan). A long lifespan gives a longer period for bioaccumulative contaminants to build up in tissues, especially in males and nonreproductive females, and for potentially higher amounts to be passed to offsprings at the start of their lives. For example, the killer and sperm whales had the highest levels of legacy POPs, which saw regulations beginning in the late 20th century, and were the only species in which DPs were found, which have been decreasingly used since 2011.^89^ It is possible that these longer-living marine mammals are reflecting previous usages of both long-regulated (e.g., legacy POPs) and recently regulated (e.g., SCCPs and DPs) contaminants.

Species-specific differences in biotransformation and elimination capacities might also contribute to the observed differences. We found both higher concentrations and relative contribution of the more persistent PCB groups I, II and V in killer whales but lower relative contribution of the more metabolizable groups IV and III, indicating elimination (Figure 2). This is despite the low δ^15^N values and trophic position of killer whales in the present study, relative to the other species (Figure 1). In contrast, harbor porpoises and humpback whales exhibited a relatively high contribution of more metabolizable PCB groups as well as the less bioaccumulative hexachlorhexanes (HCHs) (Figure 2B). While there is no data available on metabolic differences of CPs and dechloranes between marine mammal species, higher chained and chlorinated CPs are known to break down to lower chained chlorinated CPs,^90^ and differences in biotransformation abilities exist in marine mammals. For example, whales have been shown to have a reduced ability to eliminate contaminants than pinnipeds or polar bears due to differing evolutionary ancestry,^97,98^ and large interspecific differences have been observed in CYP enzyme levels among whale species due to evolutionary processes.^99^ Several studies have also reported that some species of fish appear to be better able to biotransform SCCPs than others, leading to trophic dilution and lower SCCP concentrations in predators compared to prey.^12,100^

The minke whale had the highest concentrations and proportions of MCCPs and LCCPs, but low concentrations of all other pollutants (Figures 2; 3). CPs of medium carbon chain length (Figure 4) and medium to high chlorination (Figure S5) also dominated. While samples from minke whales were obtained from harvested and presumably healthy individuals, and samples from the other species were from stranded individuals, we do not consider this a likely explanation for this large difference. We found no effect of carcass decomposition state on concentrations or patterns of contaminants in the present study (Figure 2; Table S5). Moreover, a number of minke whales present average concentrations/patterns of contaminants and overlap with the ellipses of other species. If there was a large difference between sampling the stranded and hunted individuals from, for example, differences in body condition, health, or geography, then we would expect all of the minke whales to deviate from the average. Small differences could be due to migration, which impacts pollutant exposure via prey.^99,101,102^ Most of the species in the present study remain at high latitudes throughout the year, but minke, humpback, fin, and (male) sperm whales are known to conduct long migrations from lower latitude breeding grounds to higher Arctic latitudes to feed.^70,103^ However, despite lower latitude feeding known to occur, most feeding is believed to occur in Arctic waters^103,105^ and thus there should not be large differences between contaminant levels or patterns in these species due to migration. Reasons for the higher concentrations and proportions of MCCPs and LCCPs, but low concentrations of all other pollutants in the minke whales, merit further analyses before being able to reach a conclusion.

### 3.4 Maternal Transfer

We analyzed samples from one stranded neonate killer whale, estimated to be 10 days old and still nursing, for which both legacy and emerging contaminants were previously found at similar concentrations to adults.^33^ We found SCCPs, MCCPs, and LCCPs, as well as dechlorane-602, and both DP isomers in the killer whale neonate (Table 1). Concentrations of CPs and both DP isomers were similar to those of adults, whereas concentrations of dechlorane-602 were approximately three times higher in adults (Table 1). This indicates maternal transfer of all these contaminants; however, maternal transfer factors were unable to be calculated due to no samples being available from the mother. Maternal transfer of CPs in beluga whales (*Delphinapterus leucas*) is suggested to be primarily from lactation rather than transplacental;^24^ however, SCCPs have previously been detected in the fetus of fin whales,^106^ and SCCPs, MCCPs, and LCCPs in the fetus of a minke whale,^18^ indicating some placental transfer.

There is also indication of elimination via maternal transfer in the minke whales, with the highest concentrations of all contaminants in the adult minke whale males, except LCCPs which were the highest in the subadult minke whale females (Table 1; Figure S7). The higher LCCP concentrations in the subadult females compared to adults could be from a higher ratio of LCCPs transferred from their mothers than MCCPs or SCCPs, a more efficient uptake of shorter chain CPs than longer chain CPs across a lifetime, a reduction in the use of SCCPs, and to some extent MCCPs, following regulations, or a breakdown of LCCPs to SCCPs and MCCPs throughout the lifetime.

### 3.5 Comparisons to other Studies

Lipid-adjusted concentrations of CPs in marine mammals from the present study were similar to or higher than those of other studies of marine mammals from the Arctic or subarctic (Tables 1; S8), although the aforementioned difficulties in comparing CP concentrations between laboratories should be acknowledged. Concentrations of SCCPs, MCCPs, and LCCPs in harbor porpoise were similar to that in harbor porpoise adults of both sex sampled in 2006–2012 from the Baltic Sea,^23^ and SCCP, MCCP, and LCCP concentrations were higher in all species in the present study than narwhals (*Monodon monoceros*), harbor porpoise, killer whales, and long-finned pilot whales from Greenland (Table S8).^18^ All species were also higher than beluga whales from the St Lawrence Estuary (Table S8),^24^ which is a population known for high concentrations of all contaminants.^107^ SCCP concentrations in harbor porpoises from the present study were similar to three adult harbor porpoises sampled in 2016–2018 from Sweden, but MCCP and LCCP concentrations were approximately 10 times higher (Table S8).^18^ Median concentrations of all CPs in adult male killer whales were higher than a killer whale from the Baltic Sea of the same age, class, and sex (Table S8).^18^ LCCP concentrations in the subadult female minke whales, highest in the present study, were over 30 times higher than the highest value in marine mammals found in the literature (an adult male killer whale from Sweden; Tables 1; S8).^18,23^

When comparing elsewhere, SCCP concentrations in the present humpback whales were 3–4 times higher than in nine humpback whales of mixed ages, sampled in 2007–2015 from Australia (Table S8).^108^ The highest concentrations of SCCPs and MCCPs in the present study were in an adult male sperm whale (1600 ng/g lw and 2400 ng/g lw) and were approximately half the median concentrations in marine mammals from the South China Sea (Tables 1; S8).^109^ In 2010, China was the largest producer, consumer, and exporter of CPs in the world,^110^ and higher concentrations could reflect higher local sources in China compared to Norway.

Median dechloane-602 concentrations in the killer whales were approximately 20 times higher than beluga whales of unknown age and sex from the Canadian Arctic (Table S8)^111^ and comparable to male beluga whales from the St Lawrence Estuary (Table S8).^26^ Median dechlorane-602 concentrations in minke whale of unknown age and sex from the St Lawrence Estuary were twice as high than all minke whale from the current study (Table S8).^26^

### 3.6 Thresholds for Health Effects

ΣPCB concentrations above 9000 ng/g lw have been associated with the onset of immunosuppression in harbor seals,^112,113^ and seven of the eight killer whales, three of the seven sperm whales, and one harbor porpoise exceeded this threshold. ΣPBDE concentrations above 1.5 μg/g lw have been associated with thyroid hormone disruption in gray seals pups (*Halichoerus grypus*)^114^ and one harbor porpoise and one killer whale exceeded this threshold (Table S1). Thresholds for health effects for other contaminants have not been established in marine mammals. However, while acute toxicity of CPs is generally low, chronic toxicity and sublethal effects have been reported for all CPs, including toxic effects on birds after low chronic exposure.^115,116^ MCCPs and LCCPs can furthermore break down to SCCPs and other potentially toxic and regulated compounds.^90^ A growing body of evidence indicates the potential for adverse health effects of dechloranes in a range of species,^117^ and DP has been shown to cross the blood-brain barrier in pinnipeds,^118^ which is worrying for a compound of known neurological toxicity.^119^

In this first investigation of CPs and dechloranes in a broad range of marine mammal species from Norway, we found high concentrations of these emerging contaminants. SCCPs and dechlorane-602 exhibit strong positive correlations with bioaccumulative legacy POPs. These compounds were most abundant in killer whales and sperm whales, whereas MCCPs and LCCPs, which only weakly positively correlate with the more recalcitrant legacy POPs, and were highest in minke whales. Differences in concentrations or patterns of contaminants could not be explained by dietary niche, as other large intraspecific factors may be masking effects of diet on contaminant concentrations and patterns, such as possibly lifespan or biotransformation and elimination capacities. Future studies should include other major prey or members of the coastal marine ecosystem to assess biomagnification of these contaminants. Total contaminant loads of each species were dominated by legacy contaminants and total CP loads by MCCPs, in accordance with other studies. The high concentrations and presence in a neonate killer whale provide further evidence of the bioaccumulation potential and maternal transfer of these contaminants and that MCCPs, LCCPs, and dechlorane-602 should join SCCPs in being regulated at an international level.

## Data Availability

The data sets generated during the current study are openly available in the DataverseNO repository.^120^10.18710/EHNPOW

## Supporting Information Available

Tables provide details on legacy POPs and emerging BFR concentrations, δ^13^C and δ^15^N values in the skin and muscle, limits of detection, quantification, chlorination %, and detection % of CPs, pollutant abbreviations, limits of detection, quantification, and detection % of legacy POPs and emerging BFRs, RDA model summaries, total area and proportional overlap of isotopic niches, results from Spearman’s rank correlation between contaminants, and a summary of CP and dechlorane concentrations from other studies. Figures provide sampling locations, PCA of patterns including subadult female minke whales, patterns of all organohalogen contaminants, patterns of CPs, PCA of chlorination degree, correlations between contaminants and δ^15^N values, and patterns of CPs in minke whales, and details of the method and quality assurance for the CP and dechlorane analysis (PDF)
