# Supplementary material for: Intercorrelations of Chlorinated Paraffins, Dechloranes, and Legacy Persistent Organic Pollutants in 10 Species of Marine Mammals from Norway, in Light of Dietary Niche
Source: Environ Sci Technol. 2024 Aug 9;58(33):14797–811. doi: 10.1021/acs.est.4c02625 (PMC11339914; doi:10.1021/acs.est.4c02625)
Supplement: Supplementary file 1 — es4c02625_si_001.pdf [file es4c02625_si_001.pdf]

Supporting Information for:

Intercorrelations of chlorinated paraffins, dechloranes and legacy POPs in 10 species of marine mammals from Norway, in light of isotopic niche

Clare Andvik[1*], Eve Jourdain[1,2], Anders Borgen[3], Jan Ludvig Lyche[4], Richard Karoliussen[2], Tore Haug[5], Katrine Borgå[1*]

[1] Department of Biosciences, University of Oslo, Pb 1066 Blindern, NO–0316 Oslo, Norway

[2] Norwegian Orca Survey, Breivikveien 10, NO–8480 Andenes, Norway

[3] Department of Environmental Chemistry, NILU: The Climate and Environmental Research Institute, Pb 100, NO–2027 Kjeller, Norway

[4] Department of Food Safety and Infection Biology, Norwegian University of Life Sciences, Pb 5003, NO–1432, Ås, Norway

[5] Institute of Marine Research, Fram Centre, Pb 6606 Stakkevollan, NO–9296 Tromsø, Norway

*Correspondence to: clarem@uio.no and katrine.borga@ibv.uio.no

Summary: 28 pages, 7 figures, 8 tables, 1 method text

Table of Contents

Supporting Figure S1: *Map of Scandinavia, the Barents Sea and Svalbard showing where each of the 46 marine mammals were sampled.* **...........3**

Supporting Table S1: *Median (range) concentrations of legacy POPs and emerging brominated flame retardants (PBT and HBB) in marine mammals of different species, age class and sex* **...........4**

Supporting Table S2: *$\delta^{13}C$ and $\delta^{15}N$ values in skin and muscle, and sample size, of stranded and harvested (common minke whale) marine mammals from Norway 2015–2020.* **...........5**

Supporting Method Text: *Chlorinated paraffin and dechloranes analysis* **...........6**

Supporting Table S3. *Limit of detection (LOD), limit of quantification (LOQ), mean chlorination degree, and % found in all samples of all chlorinated paraffins and dechloranes analysed* **...........8**

Supporting Table S4. *Limit of detection (LOD), mean recovery and % found in all samples of all legacy pollutants and emerging brominated flame retardants analysed.* **...........9**

Supporting Figure S2: *Principle component analysis (PCA) biplot of the patterns of homologue groups of short-, medium- and long-chain chlorinated paraffins in blubber, including sub-adult female minke whales.* **...........13**

Supporting Table S5. *Redundancy analysis model summaries.* **...........14**

Supporting Table S6: *Total area (TA), Bayesian stable isotope standard ellipse area (SEA), Bayesian stable isotope standard ellipse area corrected for small sample size (SEA_C) of four species of marine mammal sampled from coastal and Arctic Norway 2015-2020.* **...........16**

Supporting Figure S3: *Patterns of all organohalogen contaminants in blubber of 10 species of marine mammals sampled from Norway 2015-2020* **...........17**

Supporting Figure S4*: Patterns of short-, medium- and long-chain chlorinated paraffins (SCCPs, MCCPs, LCCPs, respectively) and dechloranes in blubber of 10 species of marine mammals sampled from Norway 2015-2020*...........**17**

Supporting Table S7*: Correlation coefficients (Rho) and p-values (in italics) for pairwise correlation tests between contaminants using Spearman's Rank correlation*........... **....18**

Supporting Figure S5: *Principle component analysis (PCA) biplot of the patterns of homologue groups of short-, medium- and long-chain chlorinated paraffins in blubber in nine species of marine mammal from Norway 2015–2020 (n=35). Response loadings are represented by arrows, coloured from light grey to black by increasing chlorination degree.* **...........19**

Supporting Figure S6: *The relationship between the blubber concentrations (ng/g lw) of A) ΣSCCPs B) ΣMCCPs, C) ΣLCCPs, D) Dechlorane–602 and E) PCB-153 and $\delta^{15}N$ values in skin of 9 species of marine mammal from Norway 2015–2020 (n=37)* **...........20**

Supporting Figure S7: *Patterns of short-, medium- and long-chain chlorinated paraffins (SCCPs, MCCPs, LCCPs, respectively) and dechloranes in blubber of adult male, adult female and subadult female common minke whale harvested from the Barents Sea in 2019.* **...........21**

Supporting Table S8 *Summary of chlorinated paraffin and dechlorane concentrations (ng/g lw) in blubber of other whale species referenced in the present study* **...........22**

References: **...........27**

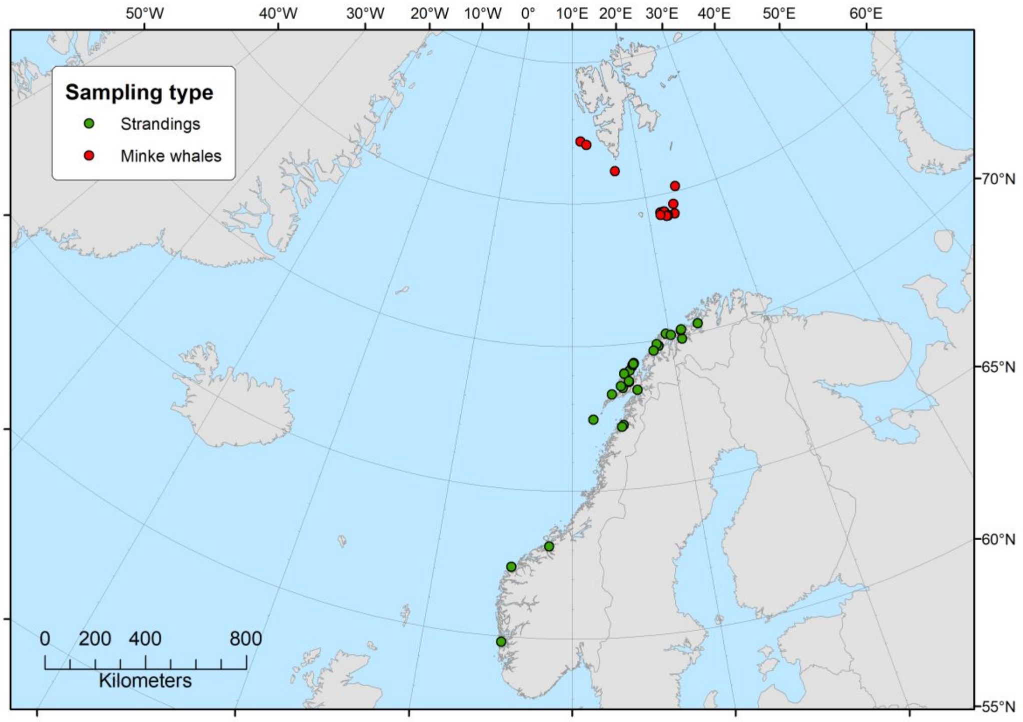

Supporting Figure S1: Map of Scandinavia, the Barents Sea and Svalbard showing where each of the 46 marine mammals were sampled. Red dots represent the 14 common minke whales obtained in 2019 from the annual harvest in the Barents Sea, and green dots the 32 marine mammals that stranded along the Norwegian coastline from 2015 to 2020.

Supporting Table S1: Median (range) concentrations of legacy POPs and emerging brominated flame retardants (PBT and HBB) in marine mammals of different species, age class and sex sampled from Norway 2015–2020 (ng/g lw). UNK= Unknown. NA= Not analyzed. LOD = Limit of detection (values given in Table S3)

Species	*n*	Age; Sex	ΣPCBs[a]	ΣDDTs[b]	ΣCHLs[c]	HCB	ΣHCHs[d]	Mirex	ΣPBDEs[e]	HBCDD	PBT	HBB
Common minke whale (*Balaenoptera acutorostrata*)	5	Adult, Female	280 (210–910)	160 (130–650)	110 (91–350)	45 (39–61)	7.7 (6.4–8.6)	5.1 (4.6–12)	11 (8.1–35)	12 (7–29)	<LOD	0.15 (<LOD–0.36)
	5	Adult, Male	3600 (720–6000)	2500 (530–4500)	1700 (390–2700)	170 (140–220)	45 (21–67)	24 (6.3–45)	72 (21–96)	25 (19–58)	<LOD	0.25 (<LOD–0.51)
	**10**	**All**	820 (210–6000)	590 (130–4500)	370 (91–2700)	100 (39–220)	15 (6.4–67)	9.2 (4.6–45)	28 (8.1–96)	22 (7–58)	0.028 (<LOD–0.041)	0.17 (<LOD–0.51)
Killer whale (*Orcinus orca*)	3	Adult, Female	20000 (18000–33000)	10000 (6600–14000)	4600 (3200–6900)	400 (300–730)	76 (64–100)	360 (210–470)	340 (260–690)	97 (<LOD–190)	0.087 (0.062–0.32)	1.7 (0.8–3.5)
	2	Adult, Male	86000 (73000–98000)	76000 (58000–93000)	15000 (9100–21000)	460 (340–580)	140 (91–180)	660 (120–1200)	1500 (1400–1700)	160 (120–190)	0.26 (0.2–0.31)	6.1 (2.2–10)
	1	Adult, UNK	15000	7100	3400	420	72	93	390	97	0.18	2.0
	1	Neonate, Male	9000	5400	2600	420	64	20	410	47	0.050	0.64
	1	Subadult, Male	7700	4600	2100	630	57	59	210	91	<LOD	1.5
	**8**	**All**	19000 (7700–98000)	8600 (4600–93000)	4000 (2100–21000)	410 (300–730)	74 (57–180)	160 (19–1200)	380 (210–1700)	97 (<LOD–190)	0.14 (0.042–0.32)	1.8 (0.58–10)
Sperm whale (*Physeter macrocephalus*)	1	Adult, Female	9900	7900	3000	270	9.0	120	150	55	<LOD	1.6
	5	Adult, Male	8300 (3200–28000)	6900 (3000–35000)	3600 (1800–13000)	550 (190–1300)	14 (9.9–47)	81 (31–250)	200 (85–590)	32 (6.9–94)	0.085 (<LOD–0.18)	0.23 (<LOD–6.6)
	1	Subadult, Male	5100	5400	2100	340	13	41	120	15	0.053	<LOD
	**7**	**All**	8300 (3200–28000)	6900 (3000–35000)	3000 (1800–13000)	390 (190–1300)	13 (9–47)	81 (31–250)	180 (85–590)	32 (6.9–94)	0.08 (<LOD–0.18)	0.23 (<LOD–6.6)
Harbor porpoise (*Phocoena phocoena*)	2	Adult, Female	32000 (3800–60000)	17000 (1900–31000)	11000 (640–22000)	3000 (150–5800)	710 (26–1400)	120 (16–220)	910 (120–1700)	1400 (110–2700)	<LOD	4.8 (1.6–7.9)
	4	UNK	2200 (1100–5100)	1300 (480–2100)	1300 (300–2300)	340 (220–490)	38 (29–42)	19 (3.1–31)	44 (35–53)	72 (31–240)	0.065 (0.043–0.098)	0.15 (<LOD–0.44)
	**6**	**All**	3400 (1100–60000)	1800 (480–31000)	1300 (300–22000)	340 (150–5800)	38 (26–1400)	24 (3.1–220)	51 (35–1700)	110 (31–2700)	0.065 (<LOD–0.098)	0.35 (<LOD–7.9)
Humpback whale (*Megaptera novaeangliae*)	1	Adult, Female	180	62	56	35	7.7	2.3	3.4	3.3	0.031	<LOD
	1	Adult, Male	220	170	150	120	23	1.3	9.3	4.0	<LOD	<LOD
	1	Subadult, Female	600	530	490	390	57	3.6	19	13	0.045	<LOD
	1	UNK	420	390	240	150	19	1.3	21	6.5	<LOD	<LOD
	**4**	**All**	320 (180–600)	280 (62–530)	200 (56–490)	140 (35–390)	21 (7.7–57)	1.8 (1.3–3.6)	14 (3.4–21)	5.2 (3.3–13)	0.024 (<LOD –0.045)	<LOD
Fin whale (*Balaenoptera physalus*)	**1**	Adult, Female	170	97	81	36	8.8	4.2	8.9	5.4	0.036	<LOD
	**1**	Adult, UNK	410	300	300	140	29	3.9	17	17	<LOD	0.13
Harbor seal (*Phoca vitulina*)	**1**	Adult, Female	640	190	100	4.9	3.5	7.3	12	2.4	<LOD	<LOD
	**2**	UNK	1100 (440 - 1800)	420 (130 - 710)	180 (70 - 290)	3.3 (3.2 - 3.4)	5.4 (2.4 - 8.4)	7.7 (5 - 10)	20 (8 - 31)	7.0 (1.7 - 12)	<LOD	<LOD
Long–finned pilot whale (*Globicephala melas*)	**1**	Subadult, UNK	7900	6400	2700	810	38	86	190	120	0.15	<LOD
Northern bottlenose whale (*Hyperoodon ampullatus*)	**1**	UNK, Male	680	530	210	52	2.6	7.8	20	4.8	<LOD	<LOD
White beaked dolphin (*Lagenorhynchus albirostris*)	**1**	UNK	7300	5500	4000	560	92	62	220	150	0.076	<LOD

[a] The sum of PCB–28, –31, –47, –52, –56, –66, –74, –87, –99, –101, –105, –110, –114, –118, –128, –137, –138, –141, –149, –151, –153, –156, –157, –170, –180, –183, –187, –189, –194, –196, –199, –206 and –209. [b] The sum of *p,p'*–DDE, *o,p'*–DDD, *p,p'*–DDD, *o,p'*–DDT and *p,p'*–DDT [c] The sum of heptachlor, oxychlordane, *trans*–chlordane, *cis*–chlordane, *trans*–nonachlor and *cis*–nonachlor [d] The sum of α–HCH, β–HCH and γ–HCH [e] The sum of BDE–28, –47, –99, –100, –153, –154, –183, –196, –202, –207, –208 and –209. PBEB and DPTE were also analyzed but under the limit of detection (LOD) in all samples.

Supporting Table S2: Median and range (in parentheses) $\delta^{13}C$ and $\delta^{15}N$ values in skin and muscle, and sample size, of stranded and harvested (common minke whale) marine mammals

Species	n (SIA skin)	n (SIA muscle)	δ^{15N} (‰)		$\delta^{13}C$ (‰)	
			Skin	Muscle	Skin	Muscle
Common minke whale (*Balaenoptera acutorostrata*)	10	10	11.6 (9.2–12.9)	11.5 (10.4–13.0)	–19.7 (–19.9—19.2)	–19.3 (–19.5—18.9)
Killer whale *(Orcinus orca)*	7	4	12.0 (11.3–14.4)	11.9 (11.7–11.9)	–19.7 (–20.5—16.4)	–19.2 (–19.6–18.8)
Sperm whale (*Physeter macrocephalus*)	6	4	14.0 (13.1–15.5)	14.1 (13.8–14.7)	–17.7 (–18.8—16.6)	–15.7 (–18.0—14.9)
Harbor porpoise (*Phocoena phocoena)*	6	5	13.3 (12.7–14.0)	12.2 (11.6–13.2)	–19.7 (–20.3—18.5)	–19.7 (–20.1–19.3)
Humpback whale (*Megaptera novaeangliae*)	3	1	12.6 (12.0–13.1)	14.6	–19.9 (–20.2—19.6)	–18.7
Harbor seal (*Phoca vitulina)*	3	2	14.4 (13.6–14.6)	14.3 (13.9–14.8)	–18.5 (–18.9—17.8)	–19.5 (-19.1—19.9)
Fin whale (*Balaenoptera physalus*)	1	2	11.6	11.8 (11.2–12.4)	–19.3	–19.1 (–19.5—18.6)
Long-finned pilot whale *(Globicephala melas)*	1	1	12.3	12.4	–17.2	–17.0
Northern bottlenose whale (*Hyperoodon ampullatus)*	0	1	NA	13.8	NA	–18.3
White-beaked dolphin (*Lagenorhynchus albirostris)*	1	1	12.2	13.1	–19.7	–19.5

from Norway 2015–2020.

Supporting Method Text: Chlorinated paraffin and dechloranes analysis

Blubber was homogenized with sodium sulphate and extracted using a cold column technique using acetone and n–hexane (1:1) with additions of internal standards (^{13}C–labelled 1,5,5,6,6,10–hexachlorodecane, dechlorane plus *syn* and dechlorane-602). Lipids were removed with sulfuric acid and the lipid content determined gravimetrically. Extracts were cleaned with 4 g activated silica and a 1 cm layer of sodium sulphate packed in a column eluted with diethyl ether in n–hexane (1:9). Solvent was changed to isooctane, volume concentrated to 100 µL, and 1,2,3,4–tetrachloronaphtalene (TCN) was added as recovery standard. The large amount of polychlorinated biphenyls (PCBs) in the samples interfered with the quantification of SCCPs and MCCPs, necessitating a fractionation by Florisil clean up. Using hexane, extracts were quantitively transferred through a pre–cleaned glass column with glass wool and burnt sodium sulphate, deactivated Florisil (1.5%) and sodium sulphate. The first elution (using hexane and dichloromethane) yields PCBs, toxaphenes and similar POPs, and the second fraction contains CPs. The second fraction was analyzed for SCCPs, MCCPs and dechloranes using gas chromatography quadrupole time–of–flight high–resolution mass spectrometry (GC/Q–TOF) (Agilent, Santa Clara, USA) in electron capture negative ionization (ECNI) mode and for LCCPs using ultra–high–performance liquid chromatography time–of–flight mass spectrometry (UHPLC–qTOF) (Agilent, Santa Clara, USA). Quantification was based on the deconvolution method developed by Bogdal et al. (2015).

Several measures were taken to reduce contamination risk during sample preparation and extraction, including using high quality solvents, burnt and solvent–rinsed glassware, and preparation procedures performed in a laminar flow hood. Where possible, samples were taken from the inside parts of the sample material to reduce the possibility of contamination from sample collection on the field. For every sample batch 1–2 blank samples were run to account for any background signal or contamination of CPs during the extraction. The fractionation by

Florisil cleanup removed the majority of confounding POPs, however toxaphene, chlordanes and nonachlor could not be removed and therefore lead to some uncertainty for SCCP and MCCP results. The LCCP analysis was unaffected by the presence of PCBs and pesticides due to differences in quantification. Values were blank corrected, and the limit of detection (LOD) was calculated as three times the standard deviation of the blank values and the limit of quantification (LOQ) as ten times the standard deviation of the blank values for each contaminant, respectively. The recovery of the CP internal standard ^{13}C–labelled 1,5,5,6,6,10–hexachlorodecane in all samples was 73 ± 15% (47–101% plus one 28%), the dechlorane internal standard dechlorane plus *syn* 56 ± 16% (25–96%). and the dechlorane internal standard dechlorane-602 43 ± 13% (21–69% plus one 12%). The LOD, LOQ, mean chlorination degree (%) and the % found in all samples can be found in Table S3.

Supporting Table S3. Limit of detection (LOD), limit of quantification (LOQ), mean chlorination degree, and % found in all samples of all chlorinated paraffins and dechloranes analysed

Pollutant	LOD (ng/g ww)	LOQ (ng/g ww)	Mean chlorination degree (%)	% found in samples
Short-chain chlorinated paraffins (SCCPs)	9.8	33	61	93
Medium-chain chlorinated paraffins (MCCPs)	3.0	10.0	53	81
Long-chain chlorinated paraffins (LCCPs)	2.5	8.3	52	95
Dibromoaldrin	0.056	NA	NA	0
Dechlorane 602	NA	NA	NA	100
Dechlorane 603	0.023	NA	NA	2
Dechlorane 604	1.3	NA	NA	0
Dechlorane 601	0.035	NA	NA	0
Dechlorane plus *syn*	0.043	NA	NA	25
Dechlorane plus *anti*	0.073	NA	NA	30

Supporting Table S4. Limit of detection (LOD), mean recovery and % found in all samples of all legacy pollutants and emerging brominated flame retardants analyzed. The laboratory is accredited by the Norwegian Accreditation for the determination of OCs and BFRs in biological matrices of animal origin according to the requirements of NSEN ISO/IEC 17025:2005 (Test 137). The laboratory is accredited for all analytes in the present study with the following exceptions: BDE-196, -202, -206, -207, -208, -209, PBT, PBEB, DPTE and HBB. The method for determining non-accredited analytes was performed and validated following the same principles as the accredited standard (NS- EN ISO/IEC 17025).

**Pollutant**	**IUPAC name**	**LOD (ng/g ww)**	**Mean recovery (%)**	**% found in samples**
PCB-28	2,4-dichloro-1-(4-chlorophenyl)benzene	0.013	85	95
PCB-31	1,4-dichloro-2-(4-chlorophenyl)benzene	0.013	88	76
PCB-47	2,4-dichloro-1-(2,4-dichlorophenyl)benzene	0.913	73	93
PCB-52	1,4-dichloro-2-(2,5 dichlorophenyl)benzene	0.017	82	100
PCB-56	1,2-dichloro-3-(3,4-dichlorophenyl)benzene	0.435	99	45
PCB-66	1,2-dichloro-4-(2,4-dichlorophenyl)benzene	0.268	104	100
PCB-74	1,2,4-trichloro-5-(4-chlorophenyl)benzene	0.594	105	100
PCB-87	1,2,3-trichloro-4-(2,5-dichlorophenyl)benzene	0.155	112	98
PCB-99	1,2,4-trichloro-5-(2,4-dichlorophenyl)benzene	0.736	107	100
PCB-101	1,2,4-trichloro-5-(2,5-dichlorophenyl)benzene	0.109	105	100
PCB-105	1,2,3-trichloro-4-(3,4-dichlorophenyl)benzene	0.029	104	100
PCB-110	1,2,4-trichloro-3-(3,4-dichlorophenyl)benzene	0.234	97	100
PCB-114	1,2,3,4-tetrachloro-5-(4-chlorophenyl)benzene	0.025	102	98
PCB-118	1,2,4-trichloro-5-(3,4-dichlorophenyl)benzene	0.025	99	100
PCB-128	1,2,3-trichloro-4-(2,3,4-trichlorophenyl)benzene	0.059	101	100
PCB-136	1,2,4-trichloro-3-(2,3,6-trichlorophenyl)benzene	3.370	70	86

PCB-137	1,2,3,4-tetrachloro-5-(2,4-dichlorophenyl)benzene	0.029	97	100
PCB-138	1,2,3-trichloro-4-(2,4,5-trichlorophenyl)benzene	0.013	95	100
PCB-141	1,2,3,4-tetrachloro-5-(2,5-dichlorophenyl)benzene	0.017	97	100
PCB-149	1,2,4-trichloro-3-(2,4,5-trichlorophenyl)benzene	0.059	100	100
PCB-151	1,2,4,5-tetrachloro-3-(2,5-dichlorophenyl)benzene	0.017	93	100
PCB-153	1,2,4-trichloro-5-(2,4,5-trichlorophenyl)benzene	0.013	91	100
PCB-156	1,2,3,4-tetrachloro-5-(3,4-dichlorophenyl)benzene	0.025	114	100
PCB-157	1,2,3-trichloro-4-(3,4,5-trichlorophenyl)benzene	0.050	119	100
PCB-170	1,2,3,4-tetrachloro-5-(2,3,4-trichlorophenyl)benzene	0.017	85	100
PCB-180	1,2,3,4-tetrachloro-5-(2,4,5-trichlorophenyl)benzene	0.029	79	100
PCB-183	1,2,3,5-tetrachloro-4-(2,4,5-trichlorophenyl)benzene	0.021	95	100
PCB-187	1,2,4,5-tetrachloro-3-(2,4,5-trichlorophenyl)benzene	0.017	91	100
PCB-189	1,2,3,4-tetrachloro-5-(3,4,5-trichlorophenyl)benzene	0.017	98	100
PCB-194	1,2,3,4-tetrachloro-5-(2,3,4,5-tetrachlorophenyl)benzene	0.017	96	100
PCB-196	1,2,3,4-tetrachloro-5-(2,3,4,6-tetrachlorophenyl)benzene	0.013	73	100
PCB-199	1,2,3,4-tetrachloro-5-(2,3,5,6-tetrachlorophenyl)benzene	0.013	69	83
PCB-206	1,2,3,4,5-pentachloro-6-(2,3,4,5-tetrachlorophenyl)benzene	0.013	101	90
PCB-209	1,2,3,4,5-pentachloro-6-(2,3,4,5,6-pentachlorophenyl)benzene	0.017	108	100
HCB	1,2,3,4,5,6-hexachlorobenzene	0.013	93	100
α-HCH	α-1,2,3,4,5,6-hexachlorocyclohexane	0.029	105	100
β-HCH	β-1,2,3,4,5,6-hexachlorocyclohexane	0.050	116	100
γ-HCH	γ-1,2,3,4,5,6-hexachlorocyclohexane	0.059	112	100
Heptachlor	1,4,5,6,7,8,8-Heptachloro-3a,4,7,7a-tetrahydro-1H-4,7-methanoindene	0.159	118	33
Oxychlordane	1,5,6,8,9,10,11,11-octachloro-4-oxatetracyclo[6.2.1.0^{2,7}.0^{3,5}]undec-9-ene	0.025	110	100

trans-Chlordane	(1S,2*S*,3*R*,4*R*,6*S*,7R)-1,3,4,7,8,9,10,10-octachlorotricyclo[5.2.1.$0^{2,6}$]dec-8-ene	0.050	114	83
cis-Chlordane	(1*R*,2*R*,3*R*,4*S*,6*S*,7*S*)-1,3,4,7,8,9,10,10-octachlortricyclo[5.2.1.$0^{2,6}$]dec-8-ene	0.067	109	100
trans-Nonachlor	(1*S*,2*R*,3*S*,5*R*,6*S*,7*R*)-1,3,4,5,7,8,9,10,10-nonachlortricyclo[5.2.1.$0^{2,6}$]dec-8-ene	0.046	118	100
cis-Nonachlor	(1*S*,2*R*,3*R*,5*S*,6*R*,7*R*)-1,3,4,5,7,8,9,10,10-nonachlortricyclo[5.2.1.$0^{2,6}$]dec-8-ene	0.025	112	100
p,p'-DDE	1-chloro-4-[2,2,2-trichloro-1-(4-chlorophenyl)ethyl]benzene	0.753	102	100
o,p'-DDD	1-chloro-2-[2,2,2-trichloro-1-(4-chlorophenyl)ethyl]benzene	0.819	108	95
p,p'-DDD	1-chloro-2-[2,2-dichloro-1-(4-chlorophenyl)ethyl]benzene	1.40	94	100
o,p'-DDT	1-chloro-4-[2,2-dichloro-1-(4-chlorophenyl)ethyl]benzene	0.978	77	98
p,p'-DDT	1-chloro-4-[2,2-dichloro-1-(4-chlorophenyl)ethenyl]benzene	0.543	190	100
Mirex	1,2,3,4,5,5,6,7,8,9,10,10-dodecachloropentacyclo[5.3.0.$0^{2,6}$.$0^{3,9}$.$0^{4,8}$]de	0.025	105	100
BDE-28	2,4-dibromo-1-(4-bromophenoxy)benzene	0.033	76	90
BDE-47	2,4-dibromo-1-(2,4-dibromophenoxy)benzene	0.067	90	100
BDE-99	1,2,4-tribromo-5-(2,4-dibromophenoxy)benzene	0.059	117	100
BDE-100	1,3,5-tribromo-2-(2,4-dibromophenoxy)benzene	0.067	104	98
BDE-153	1,2,4-tribromo-5-(2,4,5-tribromophenoxy)benzene	0.067	111	95
BDE-154	1,2,4-tribromo-5-(2,4,6-tribromophenoxy)benzene	0.067	115	100
BDE-183	1,2,3,5-tetrabromo-4-(2,4,5-tribromophenoxy)benzene	0.084	117	23
BDE-196	1,2,3,4-tetrabromo-5-(2,3,4,6-tetrabromophenoxy)benzene	0.042	80	5
BDE-202	1,2,4,5-tetrabromo-3-(2,3,5,6-tetrabromophenoxy)benzene	0.013	81	26
BDE-206	1,2,3,4,5-pentabromo-6-(2,3,4,5-tetrabromophenoxy)benzene	0.025	90	2
BDE-207	1,2,3,4,5-pentabromo-6-(2,3,4,6-tetrabromophenoxy)benzene	0.025	91	14
BDE-208	1,2,3,4,5-pentabromo-6-(2,3,5,6-tetrabromophenoxy)benzene	0.017	88	10
BDE-209	1,2,3,4,5-pentabromo-6-(2,3,4,5,6-pentabromophenoxy)benzene	0.059	102	17
HBCDD	1,2,5,6,9,10-hexabromocyclododecane	0.151	137	98

PBT	1,2,3,4,5-pentabromo-6-methylbenzene	0.021	78	48
PBEB	1,2,3,4,5-pentabromo-6-ethylbenzene	0.021	67	0
DPTE	(1,3,5-tribrom-2-(2,3 dibrompropoxy)benzene	0.167	134	0
HBB	1,2,3,4,5,6-hexabromobenzene	0.050	81	55

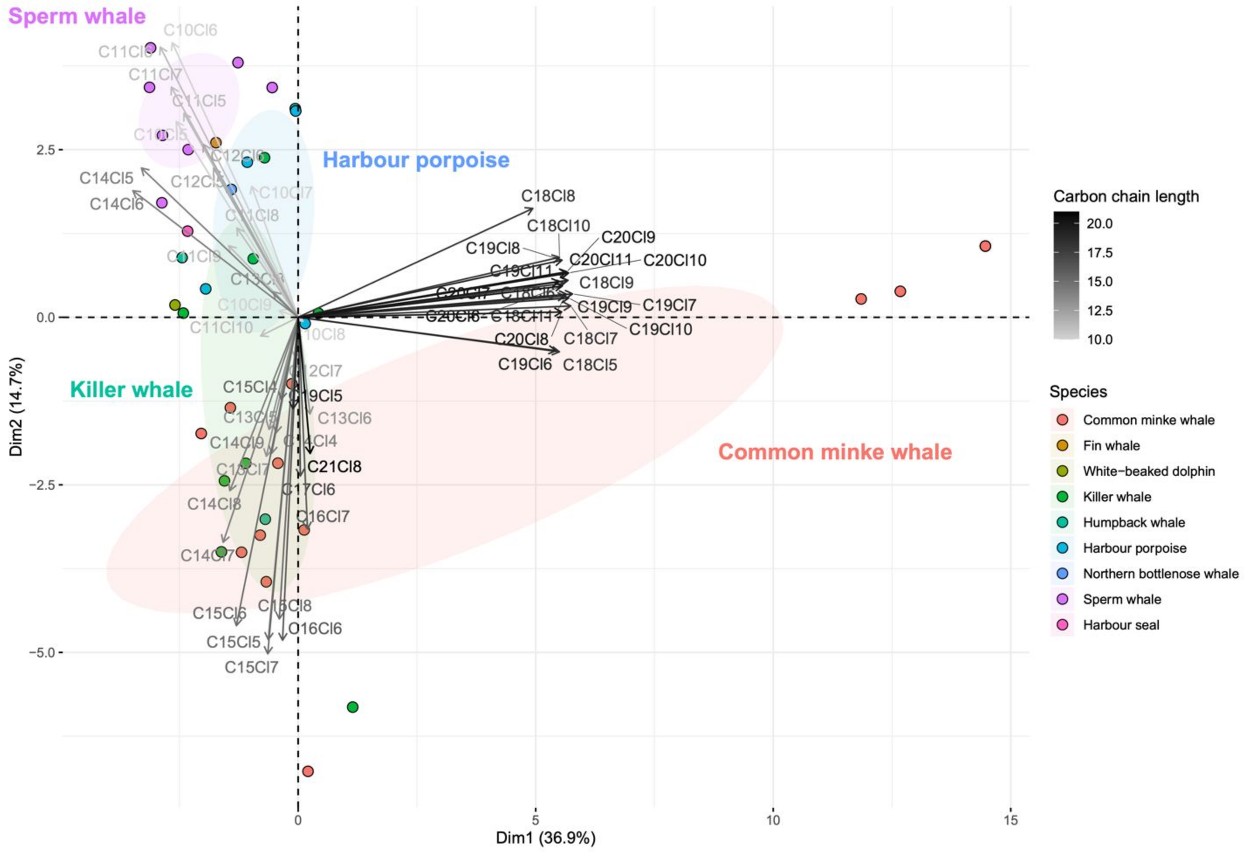

Supporting Figure S2: Principle component analysis (PCA) biplot of the patterns of homologue groups of short-, medium- and long-chain chlorinated paraffins in blubber in nine species of marine mammal from Norway 2015–2020 (n=38), including sub-adult female minke whales. Response loadings are represented by arrows, coloured from light grey to black by increasing carbon chain length. Each species are represented by unique colours. The ellipses represent 40% prediction areas for each species for multivariate normal distribution. The percentage of the total variation explained by PC1 and PC2 are given in brackets on each axis.

Supporting Table S5. Redundancy analysis model summaries. Explanatory variables present in the best model are indicated in bold, and the percentage of the total variation estimated by the full model indicated in parentheses.

With species as an explanatory variable

	Sample size	Explanatory variables	% variation explained by best model	F value best model	*P* value best model
**Concentrations**					
	42	**Species**, Lipid, Age, Sex, Decomposition	71	8.3	0.001
	37	**Species, Sex,** Lipid, Age, Decomposition, δ^{15}N in skin, δ^{13}C in skin	75 (59% Species, 4.5% Sex)	7.7	0.001
	28	**Species, Sex,** Lipid, Age, Decomposition, δ^{15}N in muscle, δ^{13}C in muscle	79 (61% Species, 8% Sex)	5.9	0.001
**Patterns**					
	42	**Species**, **Sex**, Lipid, Age, Decomposition	59 (49% Species, 5% Sex)	3.9	0.001
	37	**Species,** Lipid, Age, Sex, Decomposition, δ^{15}N in skin, δ^{13}C in skin	54	4.1	0.001
	28	**Species, Sex,** Lipid, Age, Decomposition, δ^{15}N in muscle, δ^{13}C in muscle	70 (Species 52%, Sex 9%)	3.5	0.001

Without species as an explanatory variable

	Sample size	Explanatory variables	% variation explained by best model	F value best model	*P* value best model
**Concentrations**					
	42	Lipid, Age, Sex, Decomposition	Null model	Null model	Null model
	37	**Sex,** Lipid, Age, Decomposition,	15	3.1	0.014

		$\delta^{15}N$ in skin, $\delta^{13}C$ in skin			
	28	**Sex,** Lipid, Age, Decomposition, $\delta^{15}N$ in muscle, $\delta^{13}C$ in muscle	17	2.8	0.024
**Patterns**					
	42	**Sex**, Lipid, Age, Decomposition	10	2.1	0.02
	37	**Sex**, **$\delta^{13}C$ in skin,** Lipid, Age, Sex, Decomposition, $\delta^{15}N$ in skin	20 (Sex 12%, $\delta^{13}C$ in skin 10%)	2.8	0.001
	28	**Sex. $\delta^{15}N$ in muscle,** Lipid, Age, Decomposition, $\delta^{13}C$ in muscle	26 (Sex 18%, $\delta^{15}N$ in muscle 8%)	2.8	0.001

Supporting Table S6: Total area (TA), Bayesian stable isotope standard ellipse area (SEA), Bayesian stable isotope standard ellipse area corrected for small sample size (SEA_C) of four species of marine mammal sampled from coastal and Arctic Norway 2015-2020. The probability of each niche width (SEAC) being larger than another is also indicated, as well as the proportional ellipse overlap, in a pairwise fashion.

			**A**							
			Common Minke whale Skin	Common Minke whale Muscle	Killer whale Skin	Killer whale Muscle	Harbour porpoise Skin	Harbour porpoise Muscle	Sperm whale Skin	Sperm whale Muscle
		TA	1.60	1.16	5.36	0.073	1.25	0.66	2.73	1.92
		SEA	1.00	0.55	3.94	0.13	1.00	0.55	1.93	2.12
		SEA_C	1.12	0.62	4.73	0.27	1.25	0.74	2.42	2.12
	Common Minke whale skin	**Probability that A is smaller than B / Proportional ellipse overlap**	-	0.90 / 0.42	0.0018 / 0.19	0.99 / 0.12	0.48 / 0.25	0.83 / 0.51	0.076 / 0.024	0.081 / 0.056
	Common Minke whale muscle			-	0 / 0.12	0.95 / 0.189	0.12 / 0.12	0.48 / 0.12	0.99 / 0	0.006 / 0.0091
	Killer whale skin				-	1 / 0.056	0.99 / 0.26	0.99 / 0.16	0.88 / 0.33	0.82 / 0.15
	Killer whale muscle					-	0.012 / 0.037	0.055 / 0.20	0.001 / 0	0.0018 / 0
**B**	Harbour porpoise skin						-	0.82 / 0.29	0.12 / 0.13	0.10 / 0.15
	Harbour porpoise muscle							-	0.028 / 0.058	0.027 / 0.039
	Sperm whale skin								-	0.45 / 0.33
	Sperm whale muscle									-

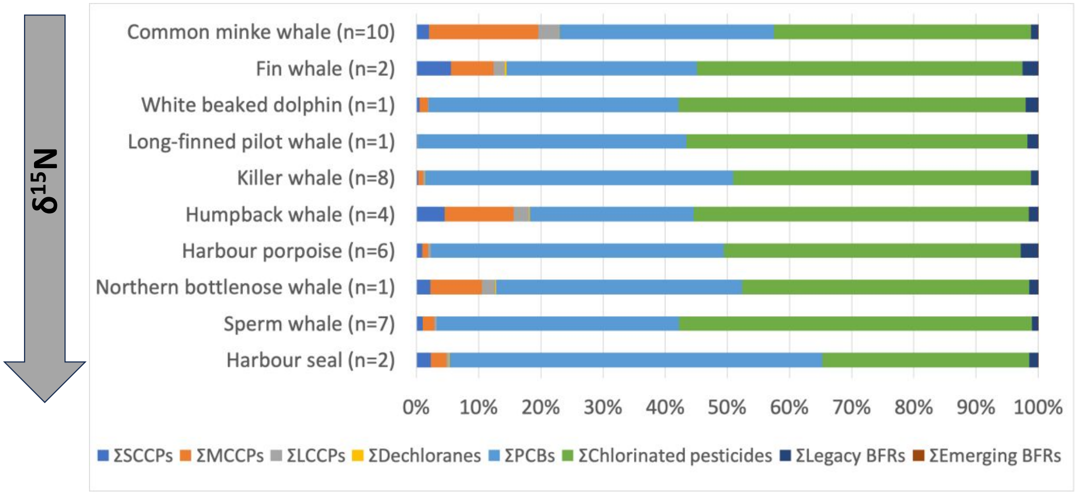

Supporting Figure S3: Patterns of all organohalogen contaminants in blubber of 10 species of marine mammals sampled from Norway 2015-2020. Species are ordered from lowest to highest mean δ15N values

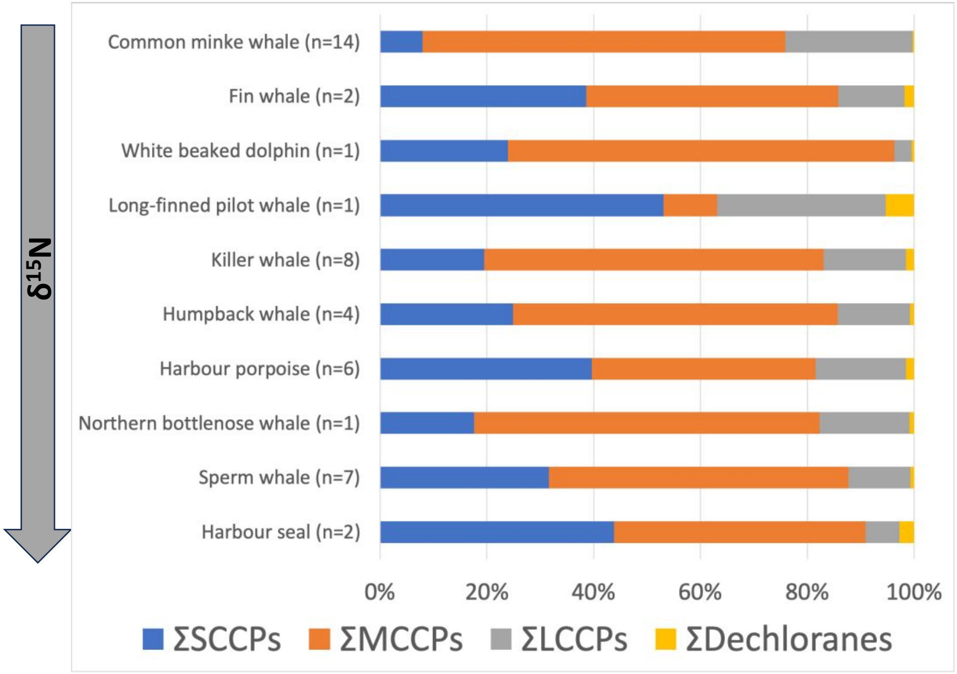

Supporting Figure S4: Patterns of short-, medium- and long-chain chlorinated paraffins (SCCPs, MCCPs, LCCPs, respectively) and dechloranes in blubber of 10 species of marine mammals sampled from Norway 2015-2020. Species are ordered from lowest to highest mean $\delta^{15}N$ values

Supporting Table S7: Correlation coefficients (Rho) and p-values (in italics) for pairwise correlation tests between contaminants using Spearman's Rank correlation. P-values are corrected for multiple testing using the Benjamini & Holmberg false discovery rate method.

	SCCPs	MCCPs	LCCPs	Dech-602	Mirex	PCB Group 1	PCB Group 2	PCB Group 3	PCB Group 4	PCB Group 5	DDTs	*trans* -chlor dane	PBDEs
SCCPs	-	0.61<br>*< 0.01*	0.45<br>*< 0.01*	0.43<br>*< 0.01*	0.50<br>*< 0.01*	0.54<br>*< 0.01*	0.54<br>*< 0.01*	0.52<br>*< 0.01*	0.49<br>*< 0.01*	0.52<br>*< 0.01*	0.55<br>*< 0.01*	-0.10<br>*0.59*	0.54<br>*< 0.01*
MCCPs		-	0.79<br>*< 0.01*	0.19<br>*0.31*	0.25<br>*0.17*	0.22<br>*0.25*	0.19<br>*0.31*	0.16<br>*0.31*	0.010<br>*0.59*	0.17<br>*0.36*	0.22<br>*0.23*	-0.32<br>*0.068*	0.20<br>*0.24*
LCCPs			-	0.22<br>*0.21*	0.23<br>*0.21*	0.18<br>*0..32*	0.14<br>*0.46*	0.13<br>*0.49*	0.071<br>*0.71*	0.15<br>*0.43*	0.15<br>*0.43*	-0.36<br>*0.03*	0.17<br>*0.33*
Dech-602				-	0.72<br>*< 0.01*	0.70<br>*< 0.01*	0.68<br>*< 0.01*	0.57<br>*< 0.01*	0.55<br>*< 0.01*	0.57<br>*< 0.01*	0.61<br>*< 0.01*	-0.24<br>*0.18*	0.58<br>*< 0.01*
Mirex					-	0.94<br>*< 0.01*	0.92<br>*< 0.01*	0.87<br>*< 0.01*	0.85<br>*< 0.01*	0.87<br>*< 0.01*	0.93<br>*< 0.01*	-0.015<br>*0.92*	0.88<br>*< 0.01*
PCB Group 1						-	0.99<br>*< 0.01*	0.90<br>*< 0.01*	0.88<br>*< 0.01*	0.95<br>*< 0.01*	0.95<br>*< 0.01*	-0.10<br>*0.59*	0.94<br>*< 0.01*
PCB Group 2							-	0.92<br>*< 0.01*	0.90<br>*< 0.01*	0.96<br>*< 0.01*	0.95<br>*< 0.01*	-0.090<br>*0.62*	0.95<br>*< 0.01*
PCB Group 3								-	0.99<br>*< 0.01*	0.94<br>*< 0.01*	0.97<br>*< 0.01*	0.04<br>*0.82*	0.95<br>*< 0.01*
PCB Group 4									-	0.94<br>*< 0.01*	0.96<br>*0.67*	0.079<br>*< 0.01*	0.95<br>*< 0.01*
PCB Group 5										-	0.96<br>*0.76*	-0.055<br>*0.90*	0.98<br>*< 0.01*
DDTs											-	0.022<br>*0.43*	0.96<br>-0.12
trans-chlordane												-	-0.12<br>*0.48*
PBDEs													-

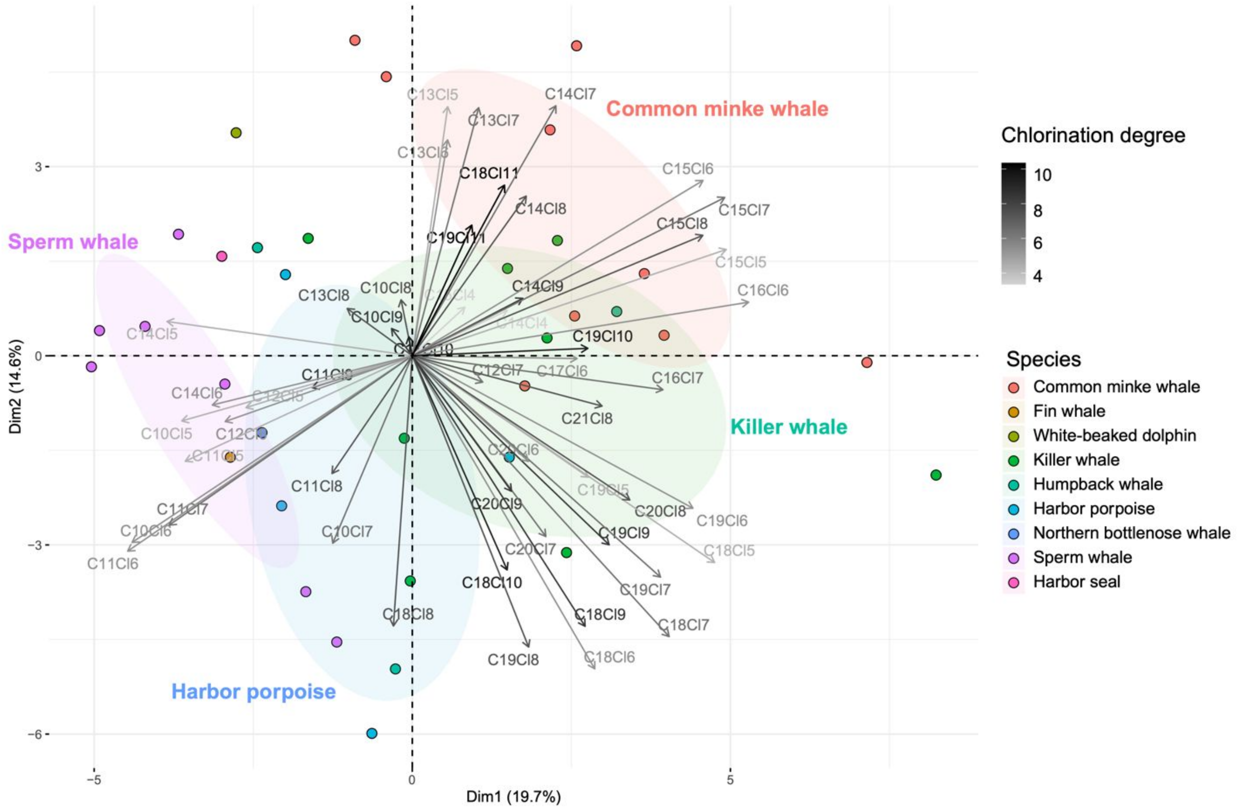

Supporting Figure S5: Principle component analysis (PCA) biplot of the patterns of homologue groups of short-, medium- and long-chain chlorinated paraffins in blubber in nine species of marine mammal from Norway 2015–2020 (n=35). Response loadings are represented by arrows, coloured from light grey to black by increasing chlorination degree. Each species are represented by unique colors. The ellipses represent 40% prediction areas for each species for multivariate normal distribution. The percentage of the total variation explained by PC1 and PC2 are given in brackets on each axis

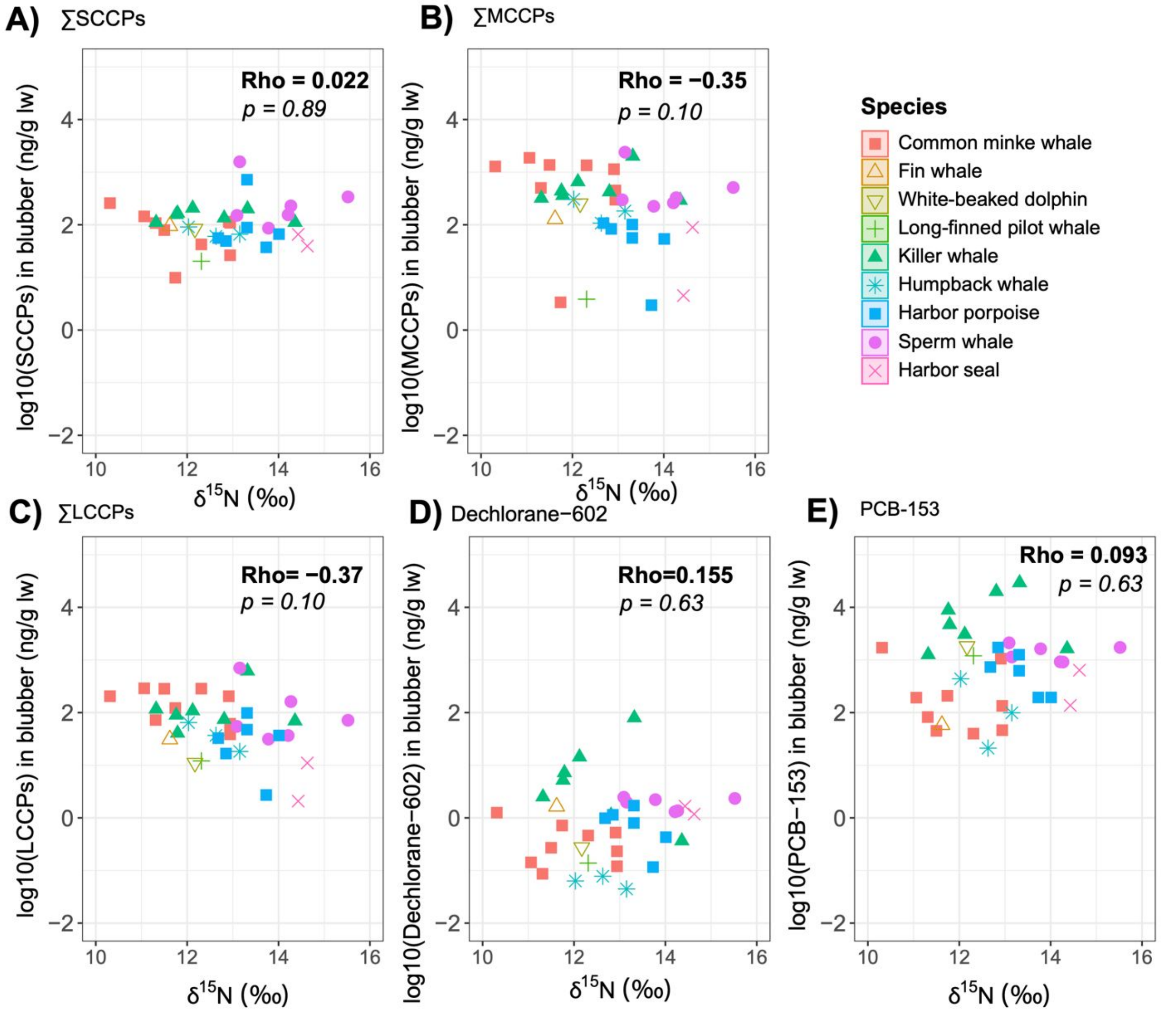

Supporting Figure S6: The relationship between the blubber concentrations (ng/g lw) of A) ΣSCCPs B) ΣMCCPs, C) ΣLCCPs, D) Dechlorane–602 and E) PCB-153 and $\delta^{15}N$ values in skin of 9 species of marine mammal from Norway 2015–2020 (n=37). The rho and p-values from Spearman's rank correlation test is indicated. P-values are corrected for multiple testing using the Benjamini & Holmberg false discovery rate method.

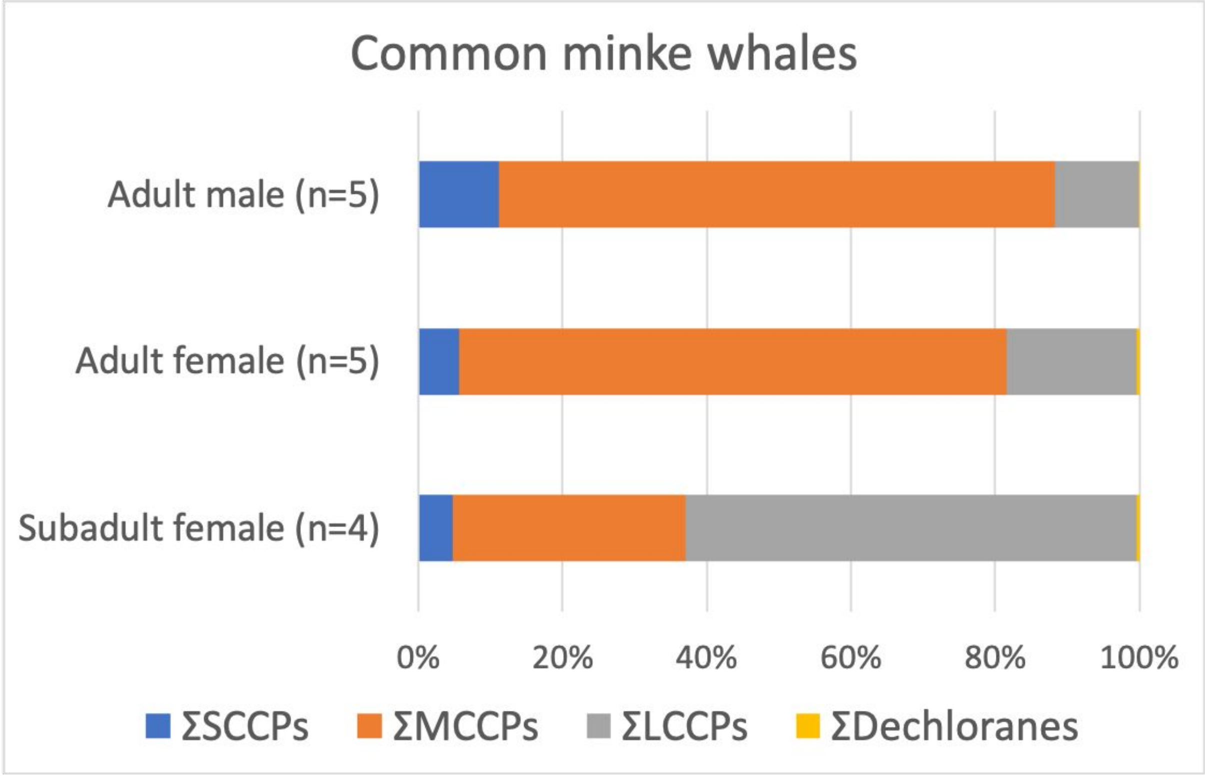

Supporting Figure S7: Patterns of short-, medium- and long-chain chlorinated paraffins (SCCPs, MCCPs, LCCPs, respectively) and dechloranes in blubber of adult male, adult female and subadult female common minke whale harvested from the Barents Sea in 2019.

Supporting Table S8 Summary of chlorinated paraffin and dechlorane concentrations (ng/g lw) in blubber of other whale species referenced in the present study. Concentrations are reported as median and range unless otherwise indicated. Studies are reported in alphabetical order by first author, and all references are reported in main text and listed in reference list.

**Species**	**Common name**	***n***	**Year sampled**	**Sex**	**Age**	**Location**	**Contaminant type**	**Median (range)**	**Reference**
Megaptera novaeangliae	Humpback whale	9	2007 - 2015	Mixed	Mixed	Australia	SCCPs	20 (< LOD – 46)	Casa et al., (2019)
Phocoena phocoena	Harbour porpoise	4 (pooled)	2008	Mixed	Adult	Baltic Sea	SCCPs	300	de Wit et al., (2020)
Phocoena phocoena	Harbour porpoise	4 (pooled)	2008	Mixed	Adult	Baltic Sea	MCCPs	36	de Wit et al., (2020)
Phocoena phocoena	Harbour porpoise	4 (pooled)	2008ß	Mixed	Adult	Baltic Sea	LCCPs	48	de Wit et al., (2020)
Phocoena phocoena	Harbour porpoise	2 (pooled)	2006 – 2012	Mixed	Adult	Baltic Sea	SCCPs	120	de Wit et al., (2020)
Phocoena phocoena	Harbour porpoise	2 (pooled)	2006 – 2012	Mixed	Adult	Baltic Sea	MCCPs	59	de Wit et al., (2020)
Phocoena phocoena	Harbour porpoise	2 (pooled)	2006 – 2012	Mixed	Adult	Baltic Sea	LCCPs	25	de Wit et al., (2020)
Phocoena phocoena	Harbour porpoise	9 (pooled)	1988-2019	UNK	UNK	Balic Sea	Dechlorane-602	1.6	Haglund & Rebryk (2022)
Phocoena phocoena	Harbour porpoise	21	2008	Mixed	Mixed	United Kingdom	Dechlorane plus *syn*	0.14 (< LOD – 0.19)	Law et al., (2013)
Phocoena phocoena	Harbour porpoise	21	2008	Mixed	Mixed	United Kingdom	Dechlorane plus *anti*	0.22 (< LOD – 0.41)	Law et al., (2013)
Delphinapterus leucas	Beluga whale	8	2000 & 2010	UNK	UNK	Canadian Arctic	Dechlorane 602	0.08 – 0.300	Shen et al., (2012)
Delphinapterus leucas	Beluga whale	4	1997 – 2013	Male	UNK	St Lawrence Estuary	Dechlorane 602	3.58 (0.22 – 7.87)	Simond et al., (2017)

Delphinapterus leucas	Beluga whale	4	1997 – 2013	Male	UNK	St Lawrence Estuary	Sum of Dechlorane *plus* and *anti*	0.44 (0.24 – 0.74)	Simond et al., (2017)
Delphinapterus leucas	Beluga whale	3	1997 – 2013	Male	UNK	Canadian Arctic	Dechlorane 602	< LOD	Simond et al., (2017)
Delphinapterus leucas	Beluga whale	3	1997 – 2013	Male	UNK	Canadian Arctic	Sum of Dechlorane *plus* and *anti*	1.28 (0.97 – 1.45)	Simond et al., (2017)
Balænoptera acutorostrata	Common minke whale	3	2002 – 2013	UNK	UNK	St Lawrence Estuary	Dechlorane 602	0.62 (0.11 – 1.65)	Simond et al., (2017)
Balænoptera acutorostrata	Common minke whale	3	2002 – 2013	UNK	UNK	St Lawrence Estuary	Sum of Dechlorane *plus* and *anti*	0.31 (0.22 – 0.42)	Simond et al., (2017)
Delphinapterus leucas	Beluga whale	5	2015 – 2019	Male	Adult	St Lawrence Estuary	SSCPs	Geomean ± SD: 29.1 ± 72.9	Simond et al., (2023)
Delphinapterus leucas	Beluga whale	5	2015 – 2019	Male	Adult	St Lawrence Estuary	MCCPs	Geomean ± SD: 24.6 ± 2.04	Simond et al., (2023)
Delphinapterus leucas	Beluga whale	7	2015 – 2019	Female	Adult	St Lawrence Estuary	SSCPs	Geomean ± SD: 11.4 ± 10.7	Simond et al., (2023)
Delphinapterus leucas	Beluga whale	7	2015 – 2019	Female	Adult	St Lawrence Estuary	MCCPs	Geomean ± SD: 3.29 ± 4.21	Simond et al., (2023)
Delphinapterus leucas	Beluga whale	5	2015 – 2019	UNK	Juvenile	St Lawrence Estuary	SSCPs	Geomean ± SD: 3.30 ± 2.03	Simond et al., (2023)
Delphinapterus leucas	Beluga whale	5	2015 – 2019	UNK	Juvenile	St Lawrence Estuary	MCCPs	Range: (< LOD - 6.10)	Simond et al., (2023)
Delphinapterus leucas	Beluga whale	4	2015 – 2019	UNK	Newborn	St Lawrence Estuary	SSCPs	Geomean ± SD: 5.15 ± 10.3	Simond et al., (2023)
Delphinapterus leucas	Beluga whale	4	1989	Mixed	UNK	NW Greenland	SCCPs	220 (110 – 250)	Tomy et al., (2000)
Phoca vitulina	Harbour seal	4 (pooled)	2012 – 2016	UNK	Adult	Scandinavian region	SCCPs	34	Yuan et al., (2019)

Phoca vitulina	Harbour seal	4 (pooled)	2012 – 2016	UNK	Adult	Scandinavian region	MCCPs	64	Yuan et al., (2019)
Phoca vitulina	Harbour seal	4 (pooled)	2012 – 2016	UNK	Adult	Scandinavian region	LCCPs	14	Yuan et al., (2019)
Phoca vitulina	Harbour seal	4 (pooled)	2006 – 2012	Mixed	Adult	Scandinavian region	SCCPs	300	Yuan et al., (2019)
Phoca vitulina	Harbour seal	4 (pooled)	2006 – 2012	Mixed	Adult	Scandinavian region	MCCPs	36	Yuan et al., (2019)
Phoca vitulina	Harbour seal	4 (pooled)	2006 – 2012	Mixed	Adult	Scandinavian region	LCCPs	48	Yuan et al., (2019)
Phocoena phocoena	Harbour porpoise	2 (pooled)	2008	Mixed	Adult	Scandinavian region	SCCPs	120	Yuan et al., (2019)
Phocoena phocoena	Harbour porpoise	2 (pooled)	2008	Mixed	Adult	Scandinavian region	MCCPs	59	Yuan et al., (2019)
Phocoena phocoena	Harbour porpoise	2 (pooled)	2008	Mixed	Adult	Scandinavian region	LCCPs	25	Yuan et al., (2019)
Monodon monoceros	Narwhal	2	2018	Male	Adult	Greenland	SCCPs	(<5.2 – 16)	Yuan et al., (2021)
Monodon monoceros	Narwhal	2	2018	Male	Adult	Greenland	MCCPs	(<10)	Yuan et al., (2021)
Monodon monoceros	Narwhal	2	2018	Male	Adult	Greenland	LCCPs	(<0.41 – 0.66)	Yuan et al., (2021)
Phocoena phocoena	Harbour porpoise	5	2018	Mixed	Mixed	Greenland	SCCPs	13 (<6.1 – 24)	Yuan et al., (2021)
Phocoena phocoena	Harbour porpoise	5	2018	Mixed	Mixed	Greenland	MCCPs	<15 (< 9.8 – 18)	Yuan et al., (2021)
Phocoena phocoena	Harbour porpoise	5	2018	Mixed	Mixed	Greenland	LCCPs	< 0.76 (<0.48 – 1.1)	Yuan et al., (2021)
Orcinus orca	Killer whale	1	2016	Female	Adult	Greenland	SCCPs	9.7	Yuan et al., (2021)
Orcinus orca	Killer whale	1	2016	Female	Adult	Greenland	MCCPs	< 14	Yuan et al., (2021)

Orcinus orca	Killer whale	1	2016	Female	Adult	Greenland	LCCPs	< 0.67	Yuan et al., (2021)
Globicephala melas	Long-finned pilot whale	3	2018	Mixed	Mixed	Greenland	SCCPs	8.3 (<5.4 – 27)	Yuan et al., (2021)
Globicephala melas	Long-finned pilot whale	3	2018	Mixed	Mixed	Greenland	MCCPs	< 12 (8.6 – 17)	Yuan et al., (2021)
Globicephala melas	Long-finned pilot whale	3	2018	Mixed	Mixed	Greenland	LCCPs	(< 0.67)	Yuan et al., (2021)
Orcinus orca	Killer whale	1	2018	Male	Adult	Sweden	SCCPs	280	Yuan et al., (2021)
Orcinus orca	Killer whale	1	2018	Male	Adult	Sweden	MCCPs	74	Yuan et al., (2021)
Orcinus orca	Killer whale	1	2018	Male	Adult	Sweden	LCCPs	32	Yuan et al., (2021)
Phocoena phocoena	Harbour porpoise	3	2016 - 2018	Mixed	Adult	Sweden	SCCPs	79 (25 – 110)	Yuan et al., (2021)
Phocoena phocoena	Harbour porpoise	3	2016 - 2018	Mixed	Adult	Sweden	MCCPs	17 (14 – 18)	Yuan et al., (2021)
Phocoena phocoena	Harbour porpoise	3	2016 - 2018	Mixed	Adult	Sweden	LCCPs	12 (11 – 13)	Yuan et al., (2021)
Neophocaena phocaenoides	Finless porpoise	50	2004 - 2014	Mixed	Adult	Hong Kong, south China	SCCPs	1800 (280 – 3900)	Zeng et al., (2015)
Neophocaena phocaenoides	Finless porpoise	50	2004 - 2014	Mixed	Adult	Hong Kong, south China	MCCPs	3200 (320 – 8600)	Zeng et al., (2015)
Sousa chinensis	Indo-Pacific humpback dolphin	25	2004 - 2014	Mixed	Adult	Hong Kong, south China	SCCPs	1900 (430 – 9100)	Zeng et al., (2015)
Sousa chinensis	Indo-Pacific	25	2004 - 2014	Mixed	Adult	Hong Kong, south China	MCCPs	4600 (530 – 23000)	Zeng et al., (2015)

	humpback dolphin								

References:

Bogdal, C.; Alsberg, T.; Diefenbacher, P. S.; Macleod, M.; Berger, U. Fast Quantification of Chlorinated Paraffins in Environmental Samples by Direct Injection High-Resolution Mass Spectrometry with Pattern Deconvolution. Analytical Chemistry 2015, 87 (5), 2852–2860.

Casà, M. V.; van Mourik, L. M.; Weijs, L.; Mueller, J.; Nash, S. B. First Detection of Short-Chain Chlorinated Paraffins (SCCPs) in Humpback Whales (Megaptera Novaeangliae) Foraging in Antarctic Waters. *Environ. Pollut.* **2019**, *250*, 953–959. https://doi.org/10.1016/J.ENVPOL.2019.04.103

de Wit, C. A.; Bossi, R.; Dietz, R.; Dreyer, A.; Faxneld, S.; Garbus, S. E.; Hellström, P.; Koschorreck, J.; Lohmann, N.; Roos, A.; Sellström, U.; Sonne, C.; Treu, G.; Vorkamp, K.; Yuan, B.; Eulaers, I. Organohalogen Compounds of Emerging Concern in Baltic Sea Biota: Levels, Biomagnification Potential and Comparisons with Legacy Contaminants. *Environ. Int.* **2020**, *144* (June), 106037. https://doi.org/10.1016/j.envint.2020.106037.

Haglund, P.; Rebryk, A. Biomagnification and Temporal Trends of New and Emerging Dechloranes and Related Transformation Products in Baltic Sea Biota. *Environ. Sci. Technol. Lett.* **2022**, *2022*, 406–412. https://doi.org/10.1021/ACS.ESTLETT.2C00171/SUPPL_FILE/EZ2C00171_SI_001.PDF

Law, Robin J., et al. "Alternative flame retardants, Dechlorane Plus and bdes in the blubber of harbour porpoises (Phocoena Phocoena) stranded or Bycaught in the UK during 2008." Environment International, vol. 60, Oct. 2013, pp. 81–88, https://doi.org/10.1016/j.envint.2013.08.009.

Shen, L.; Jobst, K. J.; Helm, P. A.; Reiner, E. J.; McCrindle, R.; Tomy, G. T.; Backus, S.; Brindle, I. D.; Marvin, C. H. Identification and Determination of the Dechlorination Products of Dechlorane 602 in Great Lakes Fish and Arctic Beluga Whales by Gas Chromatography-High Resolution Mass Spectrometry. *Anal. Bioanal. Chem.* **2012**, *404* (9), 2737–2748. https://doi.org/10.1007/s00216-012-6164-7.

Simond, A. É.; Ross, P. S.; Cabrol, J.; Lesage, V.; Lair, S.; Woudneh, M. B.; Yang, D.; Peng, H.; Colbourne, K.; Brown, T. M. Declining Concentrations of Chlorinated Paraffins in Endangered St. Lawrence Estuary Belugas (Delphinapterus Leucas): Response to Regulations or a Change in Diet? *Sci. Total Environ.* **2023**, *868* (December 2022), 161488. https://doi.org/10.1016/j.scitotenv.2023.161488.

Simond, A. É.; Houde, M.; Lesage, V.; Verreault, J. Temporal Trends of PBDEs and Emerging Flame Retardants in Belugas from the St. Lawrence Estuary (Canada) and Comparisons with Minke Whales and Canadian Arctic Belugas. *Environ. Res.* **2017**, *156* (April), 494–504. https://doi.org/10.1016/j.envres.2017.03.058.

Tomy, Gregg T., et al. "Levels of C10−c13 polychloro-n-alkanes in marine mammals from the Arctic and the St. Lawrence River Estuary." Environmental Science & Technology, vol. 34, no. 9, 17 Mar. 2000, pp. 1615–1619, https://doi.org/10.1021/es990976f.

Yuan, B.; McLachlan, M. S.; Roos, A. M.; Simon, M.; Strid, A.; Wit, C. A. de. Long-Chain Chlorinated Paraffins Have Reached the Arctic. *Environ. Sci. Technol. Lett.* **2021**, *8* (9), 753–759. https://doi.org/10.1021/ACS.ESTLETT.1C00470.

Yuan, B.; Vorkamp, K.; Roos, A. M.; Faxneld, S.; Sonne, C.; Garbus, S. E.; Lind, Y.; Eulaers, I.; Hellström, P.; Dietz, R.; Persson, S.; Bossi, R.; De Wit, C. A. Accumulation of Short-, Medium-, and Long-Chain Chlorinated Paraffins in Marine and Terrestrial Animals from Scandinavia. *Environ. Sci. Technol.* **2019**, *53* (7), 3526–3537. https://doi.org/10.1021/acs.est.8b06518.

Zeng, L.; Lam, J. C. W.; Wang, Y.; Jiang, G.; Lam, P. K. S. Temporal Trends and Pattern Changes of Short- and Medium-Chain Chlorinated Paraffins in Marine Mammals from the South China Sea over the Past Decade. Environ. Sci. Technol. 2015, 49 (19), 11348–11355. https://doi.org/10.1021/acs.est.5b02473.
